# Contrasting gene‐level signatures of selection with reproductive fitness

**DOI:** 10.1111/mec.16329

**Published:** 2022-01-17

**Authors:** Stephen J. Bush, Courtney J. Murren, Araxi O. Urrutia, Paula X. Kover

**Affiliations:** ^1^ Weatherall Institute of Molecular Medicine University of Oxford Oxford UK; ^2^ Department of Biology College of Charleston Charleston South Carolina USA; ^3^ Department of Biology and Biochemistry Milner Centre for Evolution University of Bath Bath UK; ^4^ Instituto de Ecologia UNAM Ciudad de Mexico Mexico

**Keywords:** adaptive genes, Arabidopsis, essentiality, fitness, positive selection

## Abstract

Selection leaves signatures in the DNA sequence of genes, with many test statistics devised to detect its action. While these statistics are frequently used to support hypotheses about the adaptive significance of particular genes, the effect these genes have on reproductive fitness is rarely quantified experimentally. Consequently, it is unclear how gene‐level signatures of selection are associated with empirical estimates of gene effect on fitness. Eukaryotic data sets that permit this comparison are very limited. Using the model plant *Arabidopsis thaliana*, for which these resources are available, we calculated seven gene‐level substitution and polymorphism‐based statistics commonly used to infer selection (dN/dS, NI, DOS, Tajima's *D*, Fu and Li's D*, Fay and Wu's *H*, and Zeng's E) and, using knockout lines, compared these to gene‐level estimates of effect on fitness. We found that consistent with expectations, essential genes were more likely to be classified as negatively selected. By contrast, using 379 *Arabidopsis* genes for which data was available, we found no evidence that genes predicted to be positively selected had a significantly different effect on fitness than genes evolving more neutrally. We discuss these results in the context of the analytic challenges posed by *Arabidopsis*, one of the only systems in which this study could be conducted, and advocate for examination in additional systems. These results are relevant to the evaluation of genome‐wide studies across species where experimental fitness data is unavailable, as well as highlighting an increasing need for the latter.

## 1 INTRODUCTION

Identifying genes that have undergone positive or purifying selection is a major goal in basic and applied evolutionary biology as these can reveal the molecular pathways driving adaptation to changing environments or those key to core cellular processes (Coi et al., 2017; Field et al., 2016; Good et al., 2017). Many test statistics have been devised to detect the signature of selection on protein‐coding genes or, more formally, the degree and direction of any deviation from a neutral expectation (see reviews by Biswas & Akey, 2006; Booker et al., 2017; Pavlidis & Alachiotis, 2017; Stephan, 2016). These methods, collectively known as ‘neutrality tests’, have been used very widely now that the large‐scale collection of genomic data has become affordable (Kern & Hahn, 2018; Vitti et al., 2013). The appeal of scanning the whole genome to detect genes under selection is that they take an unbiased approach to detecting the molecular basis of adaptation, without requiring prior knowledge of the nature of selection or the phenotype expected to respond.

Most neutrality tests can be classified into two main categories (Biswas & Akey, 2006; Booker et al., 2017; Pavlidis & Alachiotis, 2017; Stephan, 2016). The first category identifies a faster rate of evolution in certain genes or genomic regions when compared to a baseline (estimated by the genome‐wide rate of evolution, or the rate of evolution at synonymous sites, which are expected to be neutral or nearly neutral). This category includes the most commonly used method for detecting the signature of selection, dN/dS (Suzuki & Gojobori, 1999), as well as other estimators requiring substitution and polymorphism data such as two variants of the McDonald‐Kreitman (MK) test applied in (Stoletzki & Eyre‐Walker, 2011): “direction of selection” (DOS) and a “neutrality index” (NI), Haldane's estimator of the log‐transformed odds ratio of the MK contingency table (Haldane, 1956). The second type of test, known as a “frequency spectrum” test, aims to identify a selective sweep: the fixation of a beneficial allele under strong (positive) selection, which is characterised by a relative reduction in variation in comparison to the surrounding regions (Maynard Smith & Haigh, 1974). Tests of this category require only polymorphism data and include Tajima's *D* (Tajima, 1989), Fu and Li's D* (Fu & Li, 1993), Fay and Wu's *H* (Fay & Wu, 2000), and Zeng's *E* (Zeng et al., 2006). An overview of these methods and their interpretation is given in Supporting Information. Both types of approaches to detecting the signature of selection assume that most genes are evolving neutrally (so that those under selection can be differentiated from the baseline), an assumption that has been contested by some (e.g. Kern & Hahn, 2018; Nordborg et al., 2005).

Since every method has limitations, a multipronged approach is recommended (Vitti et al., 2013). However, because there are a limited number of species for which data is available to evaluate multiple types of signature of selection, the choice of method to detect selection is often a pragmatic one, determined by the type of sequence data available for that species and its close relatives. Methods that only require sequence divergence between species, or polymorphism data between individuals of the same species, are the most common (Nielsen, 2005).

While neutrality test statistics are frequently used to support hypotheses about the adaptive significance of particular genes, what this means phenotypically is often unclear. In the absence of supporting data, caution has previously been raised about the seductiveness of “just so” stories: superficially plausible interpretations of signatures of selection (Smith, 2016). Whole genome scans may rapidly identify (superficially) plausible candidate genes for positive selection without prior knowledge of any associated traits, but the effect these genes have on reproductive fitness is rarely tested experimentally. Generally speaking, there is a growing awareness of the need for fitness data to clarify the specific case for adaptation (Lee‐Yaw et al., 2019). Our aim with this study is to bolster this case by directly contrasting seven gene‐level signatures of selection with empirical estimates of gene effect on fitness, made using insertion mutation lines.

There are a very limited number of eukaryotic systems in which it is possible to compare multiple signatures of selection and reproductive fitness. For the purpose of this study, we use the model plant *Arabidopsis thaliana*. While *A*. *thaliana* represents short‐lived annuals, the sequence and molecular tools available to examine phenotypes in this system are more extensive than any other plant. Sequenced genomes are available both for *A*. *thaliana* (The Arabidopsis Genome Initiative, 2000) and its two sister species *A*. *lyrata* (Hu et al., 2011) and *Arabidopsis halleri* (Briskine et al., 2017), which diverged from *A*. *thaliana* approx. 5.8 Ma (Kumar et al., 2017) and 5–18 Ma (Honjo & Kudoh, 2019), respectively. In addition, genome‐wide resequencing data are available for over 1000 *A*. *thaliana* accessions from a range of ecologically diverse habitats (Cao et al., 2011; Gan et al., 2011; The 1001 Genomes Consortium, 2016). These resources enable genome‐wide scans to identify genes under positive or purifying selection using methods based on substitution and polymorphism data. Crucially, a large collection of *A*. *thaliana* knock‐out (KO) lines in a common background are available (Alonso et al., 2003; O'Malley et al., 2015), as well as quantitative fitness estimates (fruit production) for lines with a major insertion mutation in individual genes, in the form of the unPAK data set (Rutter et al., 2019). The unPAK data set differs from previous estimates of gene effects using mutant lines in other model systems (e.g. Conant & Wagner, 2004; Giaever & Nislow, 2014; Sanson et al., 2018) in that it does not address gene essentiality but whether an insertion mutation in a particular gene increases or decreases fitness measures.

Our results should be viewed as an initial foray into the problem of reconciling neutrality test statistics with direct fitness estimates at single‐gene resolution. We discuss our findings in the context of *Arabidopsis* biology and advocate for examination in additional systems. These results are relevant to the evaluation of genome‐wide studies across species where experimental fitness data is unavailable, as well as highlighting an increasing need for the latter.

## 2 MATERIALS AND METHODS

### 2.1 Sequence, variance and gene ontology annotations

CDS for *A*. *thaliana* (TAIR10), *A*. *lyrata* (v1.0) and *A*. *halleri* (Ahal2.2) were obtained from Ensembl v92 (Zerbino et al., 2018) (ftp://ftp.ensemblgenomes.org/pub/release‐38/plants/fasta/arabidopsis_thaliana/cds/Arabidopsis_thaliana.TAIR10.cds.all.fa.gz, ftp://ftp.ensemblgenomes.org/pub/release‐38/plants/fasta/arabidopsis_lyrata/cds/Arabidopsis_lyrata.v.1.0.cds.all.fa.gz, and ftp://ftp.ensemblgenomes.org/pub/release‐45/plants/fasta/arabidopsis_halleri/cds/Arabidopsis_halleri.Ahal2.2.cds.all.fa.gz, respectively, accessed 11 May 2018), as was the complete, unmasked, TAIR10 genome (ftp://ftp.ensemblgenomes.org/pub/release‐38/plants/fasta/arabidopsis_thaliana/dna/Arabidopsis_thaliana.TAIR10.dna.toplevel.fa.gz, accessed 11 May 2018).

### 2.2 Gene orthology annotation

Orthology relationships between *A*. *thaliana* and *A*. *lyrata* were obtained from Ensembl BioMart (Kinsella et al., 2011). Only genes with a reported one‐to‐one orthology with ≥75% reciprocal identity were included in analyses. Gene orthologues were further filtered based on whole‐gene dN/dS estimates, obtained from Ensembl v92 (Zerbino et al., 2018), to retain only those where 2 > dS > 0.02 and dN <2 (*n* = 17,824 genes).

### 2.3 Polymorphism data

Variant positions were obtained from three sources: a complete set of 1135 *Arabidopsis* accessions from the 1001 Genomes Project at https://1001genomes.org/data/GMI‐MPI/releases/v3.1/intersection_snp_short_indel_vcf_with_quality_reference/ (accessed 20 December 2018), and two studies with independent subsets of 19 (Gan et al., 2011) and 80 accessions (Cao et al., 2011). Variant calling was performed as previously detailed in (The 1001 Genomes Consortium, 2016). For the purposes of this study, the VCFs obtained from the 1001 Genomes Project data set are those that intersected the outcome of two variant calling pipelines, MPI (SHORE) and GMI (GATK), independently validated by the project's pilot studies (Cao et al., 2011; Long et al., 2013).

### 2.4 Tests of sequence evolution and selection

We created a data set comprising up to seven measures of sequence evolution per protein‐coding gene. Pairwise dN/dS estimates were first calculated for the coding regions of *A*. *thaliana* and (where available) its orthologue in both *A*. *lyrata* and *A*. *halleri* using the PAML package (Yang, 2007). To do so, the longest CDS of each orthologous pair was aligned end‐to‐end using the Needleman‐Wunsch algorithm, as implemented by EMBOSS needle v6.6.0 (Rice et al., 2000) with default parameters. CDS‐level dN/dS was estimated from these alignments using the Yang and Nielson model, implemented by the yn00 module of PAML v4.9h (Yang, 2007). The resulting dN/dS values were filtered to retain only those corresponding to genes with at least 75% reciprocal identity with either *A*. *lyrata* or *A*. *halleri*, 2 > dS > 0.02, and dN <2. This is because extreme dN and dS values are unreliable for estimating the dN/dS ratio and may indicate saturation with substitutions (Löytynoja & Goldman, 2008). This produces data sets of 17,630 and 8596 high‐confidence orthologues and associated dN/dS estimates, for *A*. *lyrata* and *A*. *halleri*, respectively.

A complementary set of lineage‐specific dN/dS estimates (*n* = 7086) were obtained from our previous study (Bush et al., 2015), calculated using the method of Toll‐Riera et al. (2011). This used the genome of the extremophile crucifer *Thellungiella parvula* (Dassanayake et al., 2011) as an outgroup and assumed an unrooted tree topology of ([*A*. *thaliana*, *A*. *lyrata*], *T*. *parvula*). We made multiple sequence alignments between the CDS of each *A*. *thaliana* gene, its *A*. *lyrata* orthologue (if extant) and the homologous sequence in *T*. *parvula*, using PRANK v.140110 (Löytynoja, 2014; Löytynoja & Goldman, 2008). Only those *T*. *parvula* genes with detectable homology to an *A*. *thaliana* gene for >50% of the CDS length of the longest Col‐0 transcript were used. For genes with at least 150 aligned bases, a lineage‐specific dN/dS was estimated using PAML codeml with the equilibrium codon frequencies of the model used as free parameters (CodonFreq=3). As with the pairwise dN/dS estimates, we retained only those branches showing 2 > dS > 0.02 and dN <2.

To calculate the two measures of sequence evolution that combine allele frequencies with substitutions: the neutrality index, NI (Haldane, 1956), and DOS (Stoletzki & Eyre‐Walker, 2011), we used the (Cao et al., 2011) data set with polymorphism for 80 accessions, which provide lists of SNP positions relative to the TAIR10 reference accession, Col‐0, but not their frame. To determine whether SNPs were synonymous or nonsynonymous, we first obtained the exon and UTR coordinates for each *A*. *thaliana* gene (from Ensembl v92 [Zerbino et al., 2018]), using these to derive a set of per‐transcript CDS coordinates. SNPs could then be assigned to individual codons, and their synonymous/nonsynonymous status determined. However, for genes with multiple transcripts, a large number of SNPs could be assigned to multiple sets of CDS coordinates, being read in different frames. As the calculations of NI and DOS—which are made per‐gene, not per‐transcript—require a clear and consistent distinction between synonymous and nonsynonymous polymorphisms, we excluded 1,024,068 SNPs from the Cao et al. data set as this could not be determined by this approach. We used an independent source of polymorphism data, from a study of 19 accessions (Gan et al., 2011) and which directly reports SNPs as synonymous or nonsynonymous, to ascertain this had no effect on our findings (discussed below). NI was calculated as log([2*D*_s_ + 1] [2*P*_n_ + 1]/[2*D*_n_ + 1] [2*P*_s_ + 1]) (Haldane, 1956), where *D*_n_ and *D*_s_ are the numbers of nonsilent and silent substitutions (used to calculate the pairwise dN/dS), and *P*_n_ and *P*_s_ are the numbers of nonsilent and silent polymorphisms. DOS was calculated as *D*_n_/(*D*_n_ + *D*_s_)−*P*_n_/(*P*_n_ + *P*_s_), where *D*_n_ and *D*_s_ are the numbers of nonsilent and silent substitutions, and *P*_n_ and *P*_s_ are the numbers of nonsilent and silent polymorphisms (Stoletzki & Eyre‐Walker, 2011).

Using the full set of VCFs from the 1001 Genomes Project, we calculated four additional measures of sequence evolution based on allele frequencies: Tajima's *D* (Tajima, 1989), Fu and Li's D* (Fu & Li, 1993), Zeng's *E* (Zeng et al., 2006) and Fay and Wu's *H* (Fay & Wu, 2000). These measures were calculated using the R package PopGenome v2.6.1 (Pfeifer et al., 2014) after post‐processing the set of 1135 VCFs as follows. Prior to calculation, we needed to obtain multiple sequence alignments of each CDS against the same reference genome and the same outgroup genome: *A*. *thaliana* Col‐0 (i.e., the TAIR10 accession) and *A*. *lyrata*, respectively. To do so, all variants in each VCF were first applied to the Col‐0 genome using vcf‐consensus, a component of VCFtools v0.1.16 (Danecek et al., 2011), creating one multi‐fasta file per accession. This was then partitioned into individual fasta files, one per chromosome, so that one‐to‐one whole chromosome alignments could be made between the corresponding chromosomes of each accession and Col‐0. These alignments were made using nucmer, a component of MUMmer v4.0.0beta2 (Marçais et al., 2018), with default parameters. Alignments were then parsed using the Col‐0 gene coordinates to extract, from each accession, the sequence of each gene. As *A*. *thaliana* shows extensive gene presence/absence variation (Bush et al., 2014), we confirmed that the extracted sequence corresponded to the Col‐0 gene sequence by pairwise alignment with EMBOSS needle, as above, excluding alignments with <75% identity. For each retained gene, multiple sequence alignments were then made of the *A*. *thaliana* CDS with the CDS of their *A*. *lyrata* ortholog using MAFFT v7.407 (Katoh & Standley, 2013) with default parameters, with the resulting fasta files used as input to PopGenome for *D*, D*, *E* and *H* calculation. As the power of each test depends on the number of mutations, we excluded as unreliable those estimates of *D*, D*, *E* and *H* calculated using <50 segregating sites across the multiply‐aligned CDS.

### 2.5 Gene ontology (GO) term enrichment

To assess whether any GO terms were enriched among the seven different sets of candidate genes for positive selection, gene ontology terms, and gene annotations were obtained from Ensembl BioMart (Kinsella et al., 2011). To assess GO term enrichments, we used the R package topGO v2.36.0 (http://www.bioconductor.org/packages/release/bioc/html/topGO.html, accessed 17th September 2019). topGO employs the “weight” algorithm to account for the nested structure of the GO tree (Alexa et al., 2006), and requires a user‐provided set of GO terms. For this purpose, we obtained the *Arabidopsis* TAIR10 GO annotations from BioMart (Kinsella et al., 2011) (Ensembl Plants v44), filtering them to remove GO terms with evidence codes NAS (nontraceable author statement) or ND (no biological data available), and those assigned to fewer than 10 genes in total. We retained significantly enriched GO terms (*p* < .05) only if the observed number of terms also exceeds the expected by 2‐fold or greater.

### 2.6 Testing whether essential genes show signatures of purifying selection

Essential genes should be under stronger purifying selection. We used a list of essential genes in *A. thaliana* compiled by (Lloyd et al., 2015) to assess whether they had a lower dN/dS than would be expected by chance, using a randomisation test (as in Bush et al., 2014). There are 591 essential genes with high‐confidence dN/dS estimates (out of 705 essential genes in total). Thus, subsets of 591 genes were drawn at random *s* = 10,000 times from the set of 17,630 genes for which a high‐confidence CDS‐level dN/dS estimate was available. We calculated *q*, the number of times a randomly chosen subset had a lower median dN/dS than the subset of essential genes. Letting *r* = *s−q*, then the *p*‐value of this test is *r *+ 1/*s *+ 1. This test was also applied to the distributions of DOS, NI, *D*, D*, *H*, and *E*. As the interpretation of each distribution differs, in the case of NI, *D*, D* and *E*, we instead tested whether the subset of essential genes had a higher value than expected by chance. Note also that as there are fewer estimates of *D*, D*, *H* and *E* (*n* = 15,546) than there are of dN/dS, DOS and NI (a result of filtering on the number of segregating sites; see above), there are only 549 essential genes for which *D*, D*, *H* and *E* could be estimated.

### 2.7 Gene effect on fitness

To determine the effect of different genes on fitness, we used the unPAK data set (http://arabidopsisunpak.org/, last accessed 20 November 2018) (Rutter et al., 2019). unPAK provides estimates of the total number of fruits produced by plants with different genes knocked out, as well as the ancestral wild type (accession COL70000, the background against which all KOs were made). unPAK experimental methodologies are discussed in more detail in Supporting Information, but briefly: we filtered for plants grown in growth chambers under the same controlled conditions, and only included KO lines for which there were more than three observations. This produced a set of 379 ‘unimutant’ *A*. *thaliana* lines from the Salk Institute in which the homozygous insertion of *Agrobacterium* T‐DNA is expected to knock out the gene (Alonso et al., 2003; O'Malley et al., 2015; Wang, 2008). Where available, we also obtained the ‘area ratio’, a comparison of the brightness of the PCR reaction for a single‐copy gene compared to the corresponding tDNA insertion (Rutter et al., 2017, 2019), where higher ratios indicate multiple possible insertions, that is, increased likelihood of off‐target effects. This was available as the “tdna” dataframe in the R package unpakathon v0.0.0.22 (https://github.com/stranda/unpakathon/, last accessed 20 November 2018). To estimate the relative fitness of each KO line data point in relation to the corresponding WT plant within the same experimental replicate, we divided the number of fruit (excluding aborted fruits) produced by the KO plant by the WT plant. The fitness of each KO line was then assigned as the average of all data points for each particular line. Overall, a total of 1852 fitness estimates were available, representing 379 distinct genes. A corresponding set of dN/dS, NI and DOS estimates were available in all cases, along with estimates of *D*, D*, *H* and *E* in 1665 cases (this is because allele‐frequency methods required a minimum number of segregating sites; detailed above).

## 3 RESULTS

### 3.1 Poor agreement between different molecular signatures of past selection in *A. thaliana*

**FIGURE 1:**
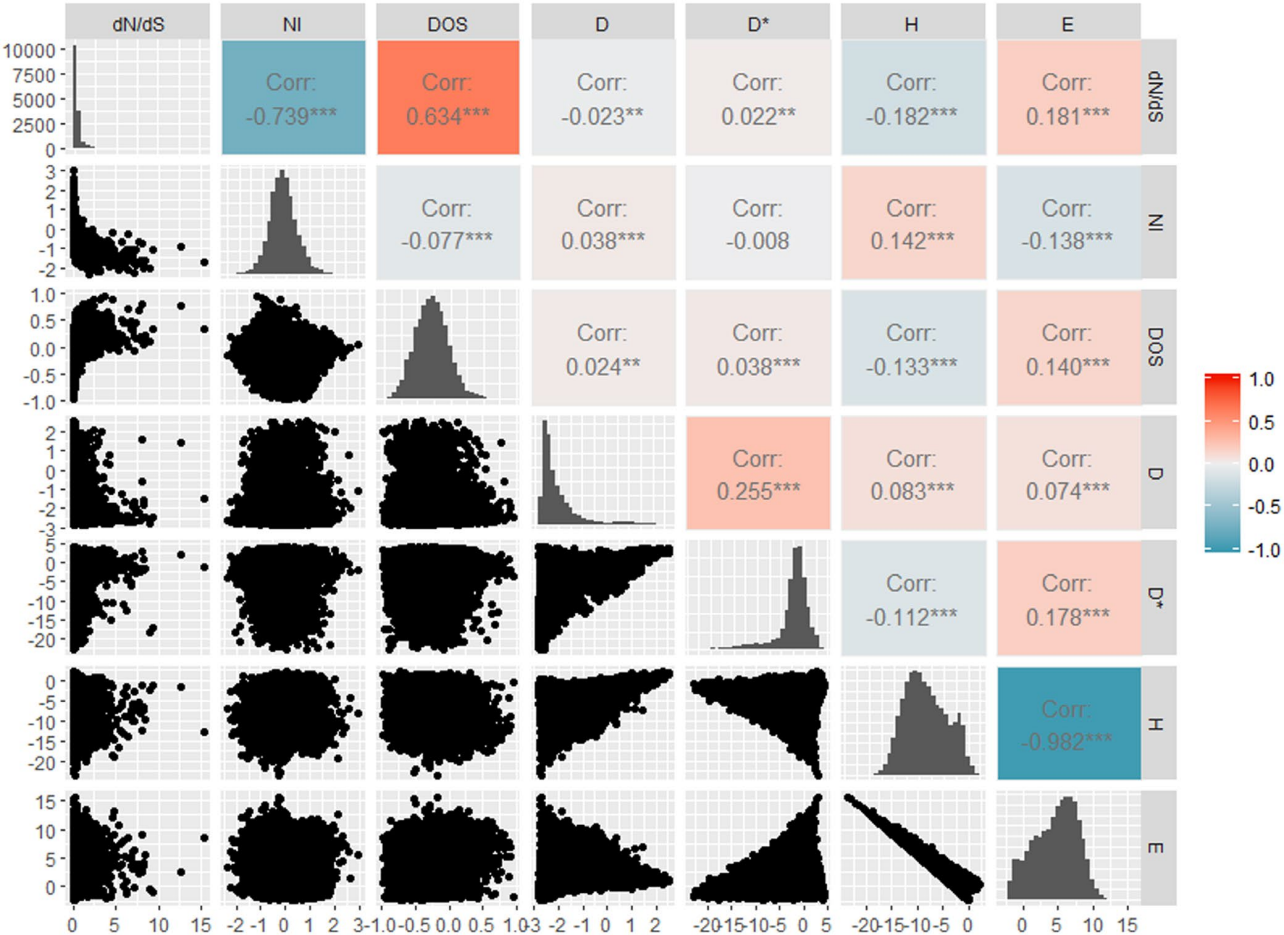
Poor agreement between different molecular signatures of past selection in *A. thaliana*. Seven estimators of past selection were calculated—*dN*/*dS*, DOS, NI, Tajima’s *D*, Fu and Li’s D*, Fay and Wu’s H and Zeng’s E (see Materials and Methods). The diagonal line of barplots shows the distribution of each estimator. The lower‐left and upper‐right plots show the correlation between each pair of estimators and the Spearman’s correlation coefficient, respectively. Significance is indicated with *(*p* < .05), **(*p* < .005) or ***(*p* < .0005). Each point represents a gene (*n* = 17,630 genes). Raw data for this figure are available in Table S1

We identified a set of 17,630 one‐to‐one *A*. *thaliana*‐*A*. *lyrata* orthologues with high reciprocal percentage identity, for which polymorphism data from subsets of 80 (Cao et al., 2011) *A*. *thaliana* accessions were collected. For these, we estimated three different substitution‐based measures of sequence evolution using CDS alignments—dN/dS, NI and DOS. For the same set of genes, we also estimated four polymorphism‐based measures of sequence evolution—Tajima's *D*, Fu and Li's D*, Fay and Wu's *H*, and Zeng's *E*—using multiply‐aligned CDS from the complete set of 1135 *Arabidopsis* accessions from the 1001 Genomes database. The complete data set of molecular signatures of past selection in *A*. *thaliana* is available as Table S1. We found only marginal similarity in gene ranking among most estimators (Figure 1). For instance, the correlation between dN/dS and Tajima's *D*, which are among the most widely used estimates, is a negligible Spearman's *rho* = –0.02. Only two estimators, Fay and Wu's *H* and Zeng's *E*, were strongly concordant (*rho* = –0.98).

### 3.2 Essential genes show molecular signatures consistent with purifying selection

A list of 705 ‘essential’ genes, whose disruption prevent the completion of the life cycle, have been compiled for *A*. *thaliana* by Lloyd et al. (2015). This set of genes should show sequence evolution signatures of purifying selection (Hurst & Smith, 1999; Wilson et al.,^1977^) and thus provide a good validation for the different methods used. Using a randomisation test, we found that, consistent with stronger purifying selection, the subset of essential genes had a higher median NI than a randomly chosen subset (*p* = 1 × 10^−4^; Table 1). These genes also had a lower median dN/dS and lower median DOS (*p* = .002; Table 1). However, there was no significant enrichment of essential genes within the distributions of the four frequency spectrum estimators (*D*, D*, *H* and *E*). We interpret this as reflecting the high sensitivity of these methods to demographic effects, which suggest they are less appropriate to detect selection in *Arabidopsis* (see Supporting Information).

**TABLE 1:** Testing whether “essential genes” show molecular signatures of purifying selection

Measure (no. of *Arabidopsis thaliana* accessions used in calculation, if applicable)	Median value for subset of essential genes	Likelihood that median value for the subset of essential genes is greater (dN/dS, DOS, *H*) or lesser (NI, *D*, D*, *E*) than the median that of a randomly chosen subset?
dN/dS (1)	0.129	1.00E‐04
NI (80)	0.067	1.00E‐04
DOS (80)	–0.324	0.002
Tajima's *D* (1135)	–2.375	0.993
Fu and Li's D* (1135)	–1.526	0.168
Fay and Wu's *H* (1135)	–7.224	1
Zeng's *E* (1135)	3.784	1

### 3.3 Identifying genes under positive selection

The number of genes identified as evolving under selection by each method, using the suggested thresholds from the literature (dN/dS > 1, DOS > 0, NI < 0, *D* < 0, D* < 0, *H* < 0 and *E *< 0; see Supporting Information) ranged from a conservative set of 923 genes with dN/dS >1 to an implausible 15,334 with Fay and Wu's *H* (Table S1) (confounding factors affecting interpretation of the tests are discussed in the Supporting Information). Summing across all methods, 16,968 genes were identified as under selection by at least one method (Table S1). However, the overlap across methods is poor, and only 29 genes are classed as under selection by all seven methods (Table S1 and Supporting Information). In addition to different methods identifying different genes, we also observe little overlap in enriched Gene Ontology (GO) biological process terms for gene sets identified as under selection by each of the seven indexes (Table S2).

### 3.4 No association between gene‐level measures of sequence evolution and reproductive fitness

**FIGURE 2:**
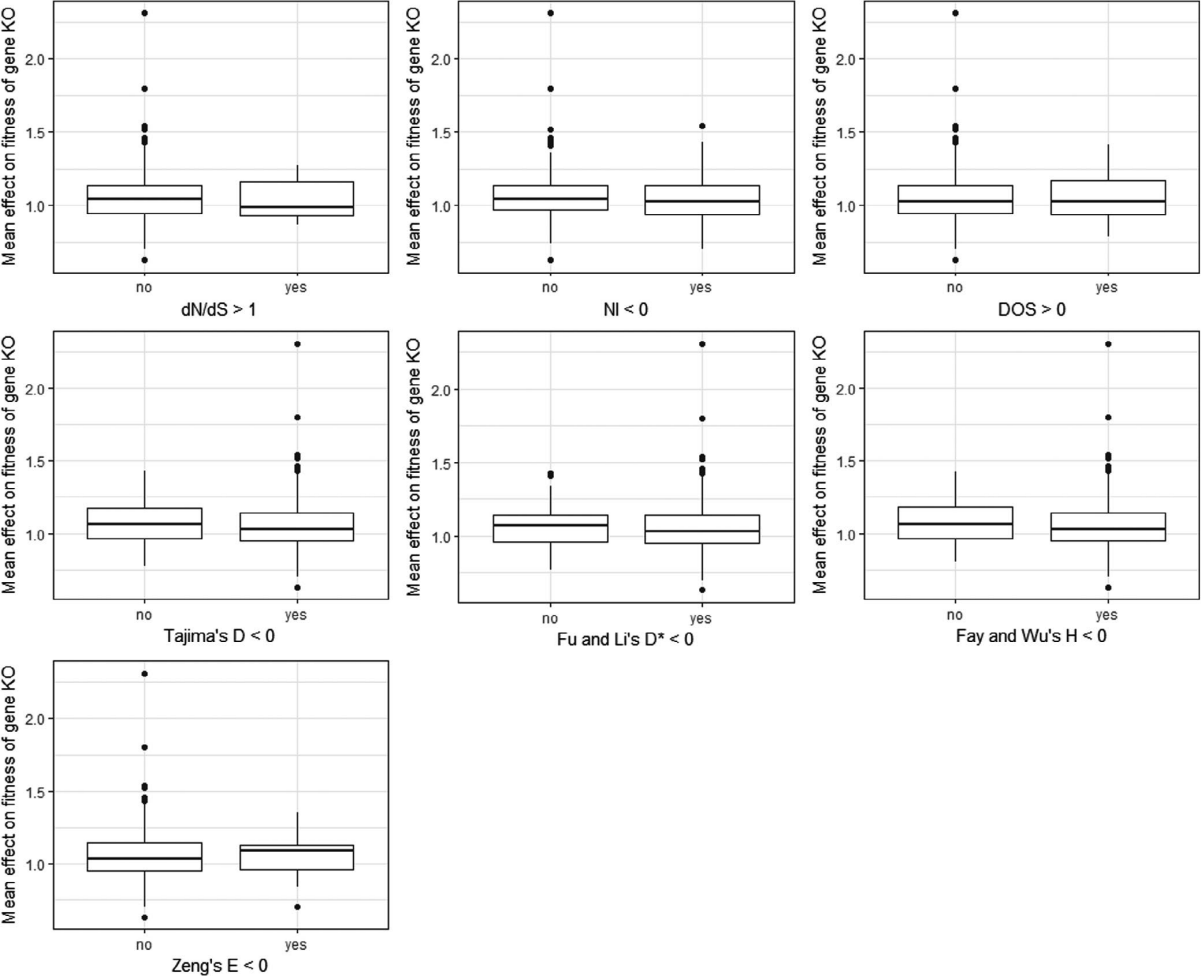
The distribution of fitness estimates does not significantly differ between genes with signatures of selection (as determined by conventional thresholds) and genes without the signature of selection. Raw data for this figure is available in Table S1. For seven different indices of sequence evolution, we used Kruskal‐Wallis tests to assess the null hypothesis that the two sets originate from the same continuous distribution. The null hypothesis was not rejected for any measure: *p* = .453 (dN/dS), 0.409 (NI), 0.716 (DOS), 0.559 (Tajima's *D*), 0.432 (Fu and Li's D*), 0.430 (Fay and Wu's *H*), and 0.906 (Zeng's *E*)

**FIGURE 3:**
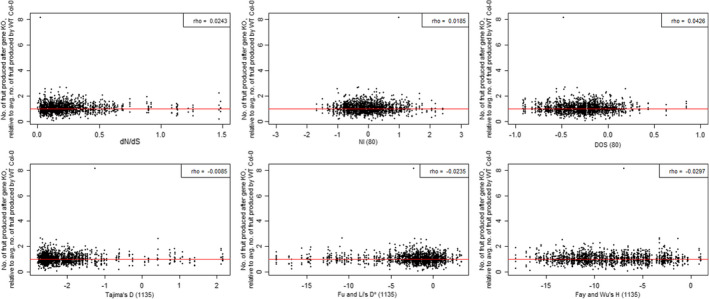
Measures of sequence evolution poorly correlate with gene effect on fitness. Contrary to the expectation that knocking out genes with strong evidence of positive or purifying selection will have a higher impact on fitness. This figure shows dN/dS, NI, DOS, *D*, D* and *H* estimates for 1852 datapoints (i.e., including all replicates for a given line) representing 379 genes. Zeng's *E*, which correlates strongly with *H* (see Supporting Information), is not shown. The upper‐right of each panel shows Spearman's *rho* for the correlations of each estimator with fitness, prior to correction for multiple testing. Although not plotted, a comparably insignificant correlation was found for Zeng's *E* (*rho* = 0.03, *p* = .22). The outlier, with a relative fitness of 8.17, is the Ras‐related protein *RABC1*, a small GTPase

We tested whether genes with signatures of selection have higher impact on fitness than genes evolving more neutrally. Fitness was estimated as the number of fruits produced by lines with insertion mutations overlapping specific genes (KO lines) relative to the fruit production of the ancestral line, which lacked them (WT line) (Table S3). Using conventional thresholds for each of the seven methods (dN/dS > 1, DOS > 0, NI < 0, *D* < 0, D* < 0, *H* < 0 and *E* < 0) we obtained sets of genes with signatures of selection, and genes without, then compared the distribution of fitness estimates between them (Figure 2). We found no significant differences in the median fitness for either of the seven sets (Kruskal‐Wallis *p* > .05 in all cases). This conclusion was robust to the use of lineage‐specific dN/dS values (using *T. parvula* [Dassanayake et al., 2011] as an outgroup to discriminate between substitutions which occurred on the *thaliana* or *lyrata* lineage; Figure S1), to the use of alternative estimates of DOS and NI (using substitution data relative to *A. halleri* and/or polymorphism data from an independent subset of 19 accessions [Gan et al., 2011]; Figure S2), and when using an outlier approach to classifying a gene as potentially positively selected, rather than conventional thresholds (considering only the top 5% of genes in each distribution; Figure S3). This conclusion was also robust to controls for population structure within the 1001 Genomes polymorphism data set (recalculating *D*, D*, *H* and *E* only using data from each of five geographically‐restricted admixture groups [171 accessions from Germany, 92 from Italy/the Balkans/the Caucasus, 64 from North Sweden, 156 from South Sweden, and 110 from Spain; groups detailed in https://1001genomes.org/accessions.html, accessed 10 February 2021]; Figure S4). In addition, the raw fitness estimates adhere closely to a normal distribution (Table S3) so their additional standardisation, as Z‐scores (to account for phenotypic variation by growth chamber), does not alter these findings (Figure S5). Further corroborating this result, we also found no significant correlations between selection estimates and the observed effect on fitness for any of the seven methods (*n* = 379 genes; Figure 3).

It is possible for KO lines to have more than one insertion site, or for unknown extra insertions to introduce fitness estimate errors. Thus, we also recalculated the correlations between selection estimates and fitness after excluding lines with an “area ratio” larger than 1.5. “Area ratio” is a comparison of the brightness of the PCR reaction for a single‐copy gene compared to the corresponding tDNA insertion, with higher ratios indicating multiple possible insertions (Rutter et al., 2019). After this filtering, we continued to find no significant correlation between sequence evolution and fitness in all seven cases (*n* = 236 genes; Figure S6).

## 4 DISCUSSION

In this study, we examined the relationship between various “neutrality tests” commonly used across evolutionary systems to identify genes under selection, and empirical estimates of gene effect on fitness. Previous studies have found an association between genes that are essential and genes that are identified as evolving under purifying selection—but how signatures of positive selection relate to empirical assessments of a gene's effect on fitness is rarely considered, given the difficulty in measuring fitness experimentally. To the best of our knowledge this is the first systematic attempt to examine the relationship between signatures of positive selection and empirical measures of fitness.

### 4.1 Fitness effects of genes with signatures of selection

After calculating seven different neutrality tests, we found that neutrality tests are more successful at identifying genes that are essential (for which a knockout will be lethal) than genes that have beneficial effects (those where a knockout will reduce fitness). This might be because beneficial and deleterious traits have very different mutation profiles, and that neutrality tests fit negative selection better. However, it is difficult to evaluate this hypothesis as both the relative occurrence of different types of mutation, as well as the distribution of their fitness effects, remain poorly understood (Berdan et al., 2021). SNPs (the most common type of mutation) are most often deleterious and purged through negative selection as envisioned by neutrality tests (Keightley & Eyre‐Walker, 2010). In contrast, while the most likely path to adaptation remains unknown, mutations other than SNPs are important in the adaptive process (Berdan et al., 2021)—although it is currently much easier to detect single nucleotide substitutions with the present resequencing techniques than insertions, deletions and rearrangements (Ho et al., 2020). More importantly, most neutrality tests are designed to detect positive selection on rare gain‐of‐function alleles that sweep through populations quickly, which might not be the most common process through which adaptations occur. For example, selection on quantitative traits, especially when relying on standing genetic variation, is likely to be operating on multiple combinations of alleles simultaneously, and therefore not show the expected patterns of complete substitution or linkage disequilibria expected with fast fixation. Equally, genes experiencing adaptive loss‐of‐function are likely to be heterogeneous, violating the core assumptions of traditional neutrality tests (Pennings & Hermisson, 2006).

Insertion mutations are usually assumed to cause loss of function in the genes they occur, and therefore to be deleterious. Thus, it might be surprising to see that twice as many genes for which the mean effect of an insertion mutation on fitness is >1 than <1 (*n* = 233 and 142 genes, respectively, as detailed in Table S3; note there are four genes where KO effect on fitness is equal to 1). However, evidence has been accumulating for nonfunctional alleles to significantly contribute to adaptation (Monroe et al., 2021). Also, the fact that gene presence/absence variation is pervasive across plant species (Bayer et al., 2020), including *A*. *thaliana* (Bush et al., 2014), support the idea that loss of function can be associated with an increase in fitness. In our study, it is possible that some of the loss of function associated with increased fitness is due to the experiment being carried out under controlled and well‐provisioned conditions, but is worth noting that similar results were also observed for insertion‐mutation and mutation‐accumulation lines grown in field conditions (Rutter et al., 2018, 2019).

Our results raise some important questions about the relationship between neutrality tests and their ability to identify genes potentially involved in adaptive evolution. However, because estimating gene‐level fitness effects remains challenging given the difficulty in performing functional genomics experiments at large scale, these results must be taken very carefully, as we discuss below. Our inability to detect an association emphasizes the importance of exploring different approaches to empirically validating signatures of selection, and how to do so at a larger scale.

### 4.2 Different signatures of selection provide discordant results

In general, we found poor agreement between different neutrality tests. Such a pattern has also been reported in humans (Oleksyk et al., 2010) and in the plant *Medicago trunculata* (Paape et al., 2013), indicating that this may be a general pattern across eukaryotes. The fact that different methods identified different genes as under selection may not be surprising because each method relies on evidence from different evolutionary time scales (Sabeti et al., 2006). However, there is little clarity about what time period each method explicitly targets and as it is unlikely that any particular neutrality test is specific to a distinct period in time, a degree of overlap should be seen. Thus, while differences in the temporal specificity of different methods can explain some of the discrepancy, it is unlikely to be the main explanation for the very low correlation between methods.

The disagreement between neutrality tests may also partly arise from the fact that some of the methods commonly used are vulnerable to demographic effects, particularly those drawing on frequency spectrum data (such as *D*, D*, *H* and *E*; see Supporting Information and references therein). Thus, it is possible that many of the genes identified as being under selection are false positives, and therefore not replicated across methods. In our data set, the influence of nonselective forces was particularly apparent when estimating Fay and Wu's *H*, which showed a whole genome distribution skewed towards negative values (Figure 1 and Supporting Information), attributable to inbreeding (Abbott & Gomes, 1989; Agrawal & Hartfield, 2016; Bergelson et al., 1998; Marais et al.,^2004^; Platt et al., 2010; Schmid et al., 2006; Tyagi et al., 2015).

Despite the general lack of agreement between methods, we were still able to identify 29 genes that are supported as under positive selection by all seven signatures of selection employed here. Unfortunately, KO lines for these 29 genes were not included in the unPAK data set, so we could not determine fitness impact of these genes. We were also unable to find previous studies that considered the effect of these genes. Future studies specifically examining the possible fitness effects of these genes are needed.

### 4.3 Important caveats of this work

To the best of our knowledge, *A*. *thaliana* is one of the very few species with comprehensive gene‐level substitution, polymorphism, and fitness effect data available to perform the tests carried out here. Nevertheless, this impressive collection of data is still far from an ideal data set to make a final conclusion about the relationship between signatures of selection and beneficial fitness effects, and we must consider three important caveats:

Firstly, *A*. *thaliana* presents its own analytic challenge as, being a selfer, its set of neutrality test statistics is particularly influenced by demographic effects. This is especially apparent with Fay and Wu's *H*, which showed a whole genome distribution skewed towards negative values, a well‐known consequence of inbreeding (Abbott & Gomes, 1989; Agrawal & Hartfield, 2016; Bergelson et al., 1998; Marais et al., 2004; Platt et al., 2010; Schmid et al., 2006; Tyagi et al., 2015). It is important to stress that our conclusions with regard to the poor agreement between each measure and reproductive fitness nevertheless do not rely on individual measures—accepting that some measures may not give a clear signal in *A*. *thaliana*, especially those based on frequency spectra—but apply to every measure tested. We may exclude data from any of the seven measures and still draw the same conclusion.

Secondly, there are still a number of limitations in estimating the fitness impact of individual genes. The lack of agreement between fitness estimates from the unPAK data set and signatures of selection may be due to the fact that a “KO” for a given gene may not necessarily correspond to a line where that gene is not functional, or where the targeted gene is the only one knocked‐out. These *Arabidopsis* mutant lines are created by Ti insertion (Alonso et al., 2003; O'Malley et al., 2015) and depending on where the insertion occurs it may just alter the protein instead of completely preventing its expression. It is also possible that insertions occur in more than one location. In this study, the latter problem has been mitigated by selecting only for lines of *A*. *thaliana* in which there are homozygous insertions of *Agrobacterium* T‐DNA into target genes (Alonso et al., 2003; O'Malley et al., 2015; Wang, 2008). However, for a number of genes, it remains uncertain that T‐DNA insertion has necessarily resulted in complete silencing (Jupe et al., 2019) as silencing may be sensitive to the insertion position (Murren et al., 2019; Valentine et al., 2012). In addition, the KO lines used here all were produced in a single genetic background (Col‐0), which may itself impact the fitness effect estimates of any gene silenced. Taken together, this suggests that there is a need for additional functional characterization of each KO line, although this would represent a substantial amount of work for large screens. Such refined studies are in any case only currently possible in experimental systems such as *A. thaliana*, where extensive tools are available.

It is also possible that fruit count under a controlled environment (as used in the unPAK data set) might not be the closest proxy for fitness. Although we found that fruit production is significantly correlated with biomass, inflorescence height, number of rosette leaves, and rosette diameter (Figure S7)—collectively suggesting that plants with a higher fruit count are, in general, fitter—we must acknowledge that the estimate of fitness used here was restricted to a single environmental condition, which may not be appropriate to detect fitness consequences associated with a specific environment. For example, positively selected pathogen‐associated genes would not necessarily have shown a decrease in fitness when knocked out under the unPAK conditions, since the necessary pathogen was not present. Similarly, genes associated with an increase in fitness many generations ago may still show a strong molecular signature of selection but no longer affect fitness in the present environment. Nevertheless, the broad associations described here are not sensitive to specific genes that only function in one environment. It is also not practically possible to exclude individual genes from the analysis on the basis that there is a particular reason why knocking them out no longer matters in this environment, although we cannot rule out that this explanation may suffice for a proportion of them.

Lastly, we must acknowledge that we were limited in terms of sample size to the 379 genes (approximately 1.4% of the total of 27,655 annotated genes) for which fitness estimates were available. It is unclear whether this is a representative sample, particularly as outliers would be expected in *Arabidopsis*: a number of genes experience adaptive loss‐of‐function and so would violate the assumptions underlying the neutrality tests used here (Monroe et al., 2021).

In conclusion, and mindful of the caveats above, we do not find a relationship between gene‐level signatures of selection and effect on fitness in our *Arabidopsis* data set. We approach these results with caution and conclude that the abundance of gene‐level neutrality test statistics is far from matched by a complementary set of fitness estimates. We believe our study clearly highlights the need for increased efforts in estimating gene‐level fitness using other technologies (such as NILS and CRISPR) to characterize the fitness effects of genes under many environments, both in *A*. *thaliana* (an important model system for most plant crops) and other species (as recently advocated by Kerwin et al., 2015; Lee et al., 2014)).

## CONFLICT OF INTEREST

The authors have no conflict of interest to declare.

## AUTHOR CONTRIBUTIONS

PXK, SB, CM & AUO Conceived and designed the study, contributed data and wrote the paper. SB Performed data analysis.

## Supporting information

Fig S1‐S5

Table S1‐S3

Text S1

## Data Availability

*Data availability*: The processed data underlying this article are available in its online Supporting Information material. Raw data are available via the 1001 Genomes Project (www.1001genomes.org) with accession metadata detailed at https://1001genomes.org/accessions.html. The scripts used to calculate each neutrality test are available at www.github.com/sjbush/athal_selection. *Benefit*‐*sharing*: Benefits from this research accrue from the public sharing of our data and code, as described above.
