# Supplementary material for: Contrasting gene‐level signatures of selection with reproductive fitness
Source: Mol Ecol. 2022 Jan 17;31(5):1515–26. doi: 10.1111/mec.16329 (PMC9304172; doi:10.1111/mec.16329)
Supplement: Supplementary file 1 — Fig S1‐S5 [file MEC-31-1515-s001.docx]

**Supplementary Figures**


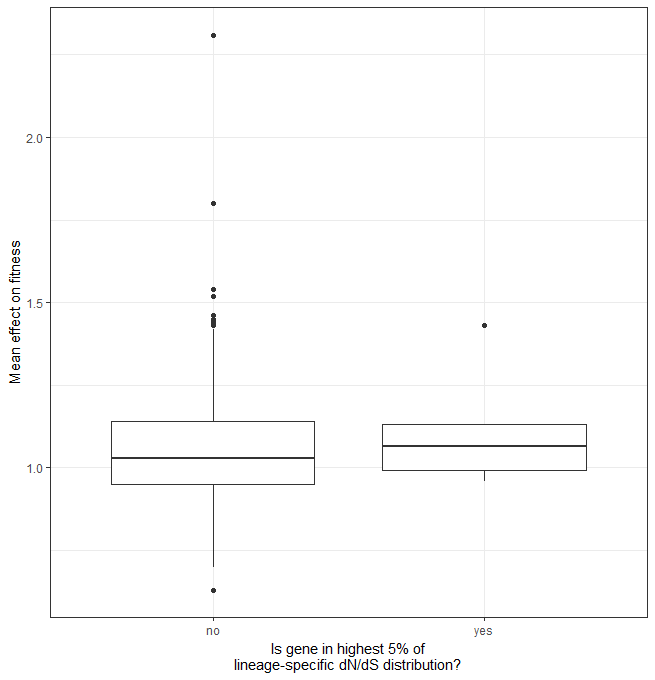


**Supplementary Figure 1.** The distribution of fitness estimates does not significantly differ between genes with signatures of selection (considered to be the top 5% of genes in the distribution), and genes without the signature of selection.

Lineage-specific dN/dS data was obtained from a previous study (Bush, Kover, & Urrutia, 2015). Rather than using a conventional threshold of dN/dS > 1, for the purpose of this figure, genes were considered positively selected on the basis of the top 5% of genes in the distribution (i.e. dN/dS > 0.34). This was because there was otherwise no overlap between the set of genes with dN/dS > 1 and the set of genes with fitness estimates. A Kruskal-Wallis test to assess the null hypothesis that the two sets originate from the same continuous distribution was rejected (p = 0.5527).


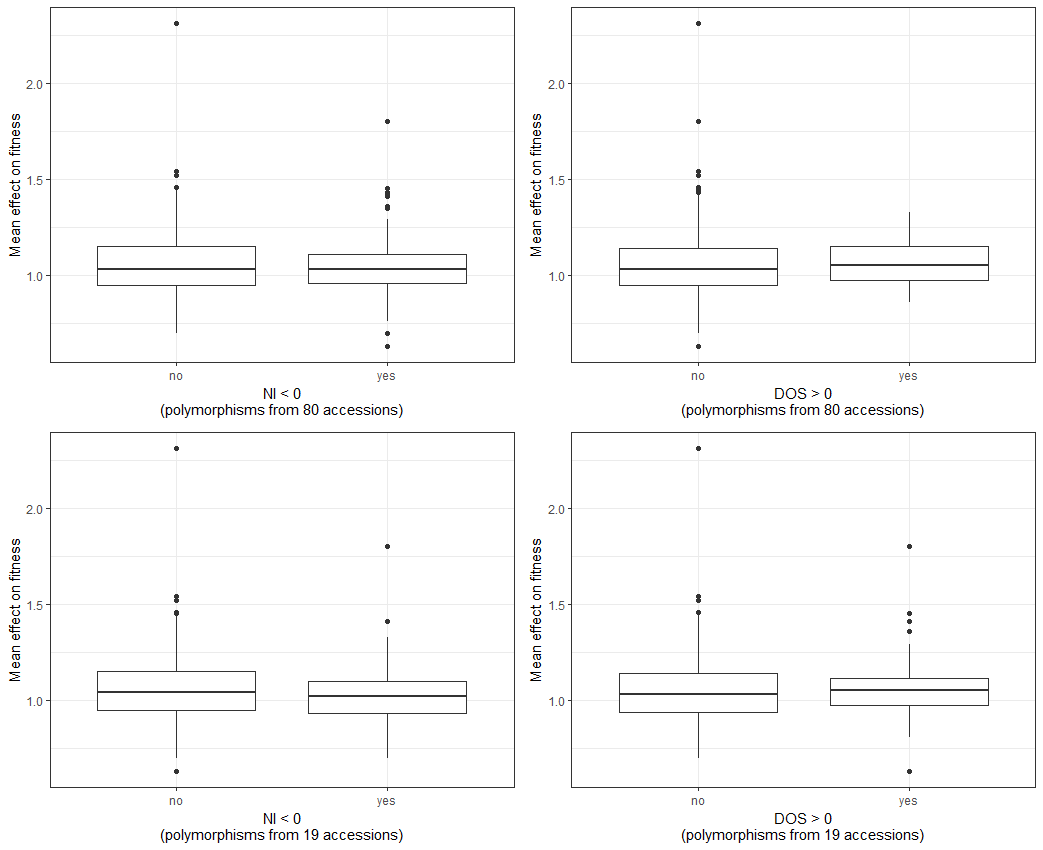


**Supplementary Figure 2.** The distribution of fitness estimates does not significantly differ between genes with signatures of selection (as determined by conventional thresholds for DOS and NI), and genes without the signature of selection, when using alternative data sources: substitution data derived from *A. halleri* rather than *A. lyrata*, and polymorphism data derived from a set of 19 *A. thaliana* accessions (Gan et al., 2011).

Raw data for this figure is available in **Supplementary Table 1**. This figure complements **Figure 1**, which shows the distribution of fitness estimates for genes considered positively selected on the basis of substitution data derived by CDS alignment against *A. lyrata*. This figure shows the equivalent values of NI and DOS, re-calculated using substation data derived from CDS alignments against *A. halleri* and/or with an alternative set of polymorphism data. For each index of sequence evolution, we used Kruskal-Wallis tests to assess the null hypothesis that the two sets originate from the same continuous distribution. The null hypothesis was not rejected for any measure. Using polymorphism data from 80 accessions, p = 0.6558 (NI) and 0.6545 (DOS), and from 19 accessions, p = 0.1309 (NI) and 0.8336 (DOS).


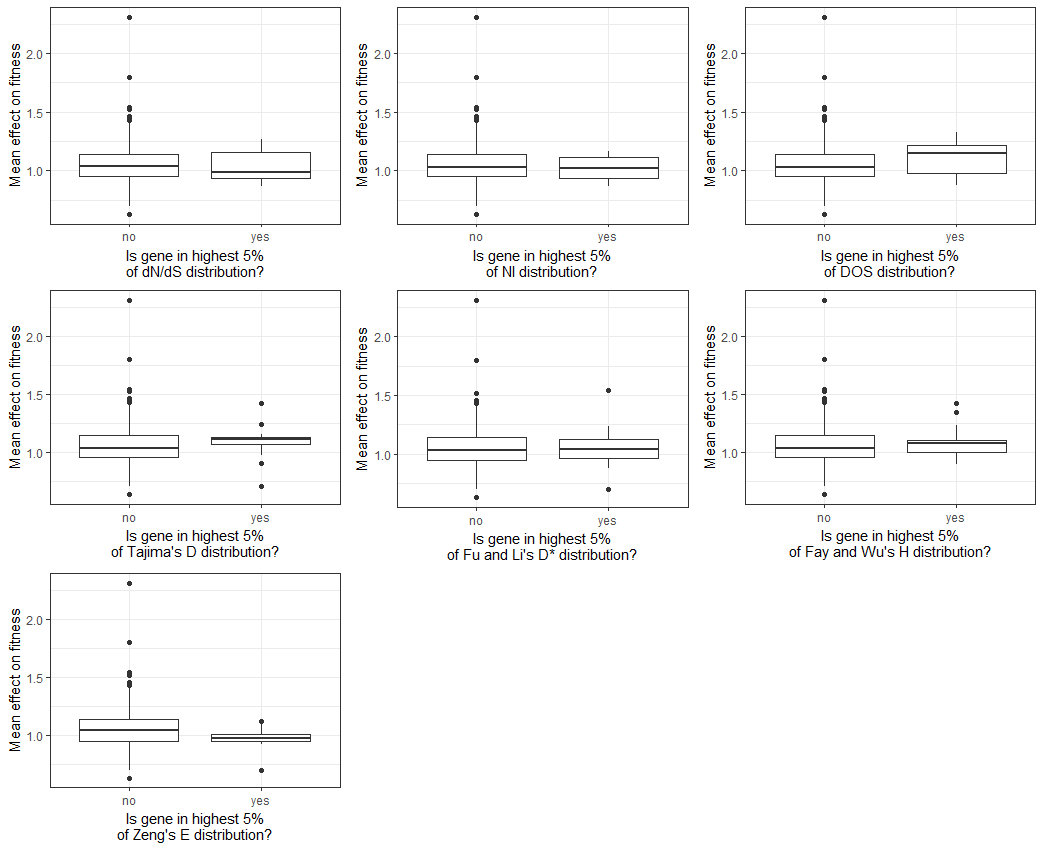


**Supplementary Figure 3.** The distribution of fitness estimates does not significantly differ between genes with signatures of selection (considered to be the top 5% of genes in the respective distribution), and genes without the signature of selection.

Raw data for this figure is available in **Supplementary Table 1**. For seven different indices of sequence evolution, we used Kruskal-Wallis tests to assess the null hypothesis that the two sets originate from the same continuous distribution. The null hypothesis was not rejected for any measure: p = 0.4534 (dN/dS), 0.4262 (NI), 0.2286 (DOS), 0.1673 (Tajima’s D), 0.9965 (Fu and Li’s D*), 0.4383 (Fay and Wu’s H), and 0.08665 (Zeng’s E).


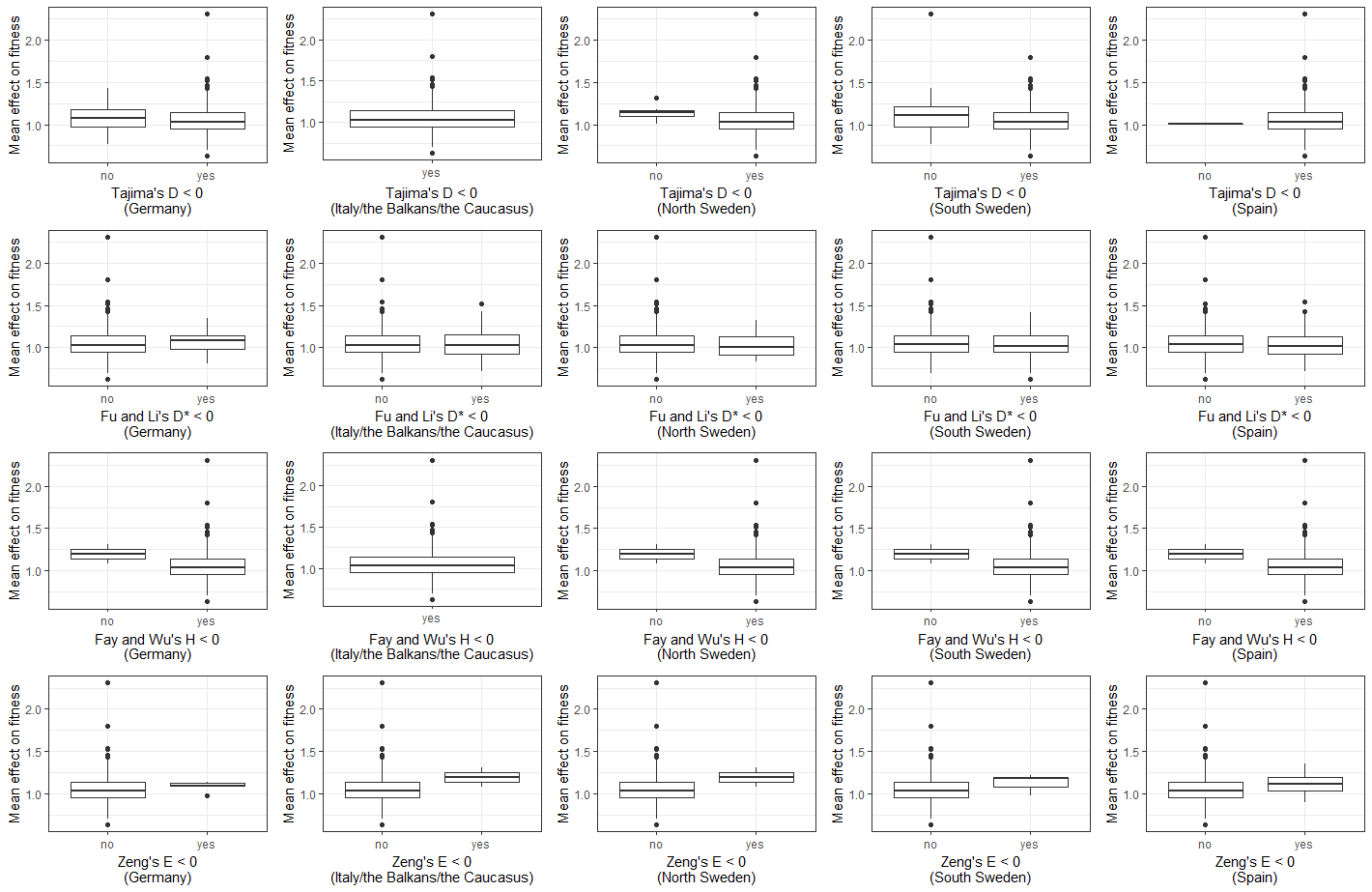


**Supplementary Figure 4.** The distribution of fitness estimates does not significantly differ between genes with polymorphism-based signatures of selection (as determined by conventional thresholds for Tajima’s *D*, Fu and Li’s *D**, Fay and Wu’s *H*, and Zeng’s *E*), and genes without the signature of selection, after controlling for population structure.

Raw data for this figure is available in **Supplementary Table 1**. This figure complements **Figure 1**, which shows the distribution of fitness estimates for genes considered positively selected on the basis of *D*, *D**, *H*, and *E*, using the full dataset of 1135 *Arabidopsis* accessions. This figure shows the re-calculation of these values when using data only from 5 geographically-restricted admixture groups: 171 accessions from Germany, 92 from Italy/the Balkans/the Caucasus, 64 from North Sweden, 156 from South Sweden, and 110 from Spain (groups detailed in https://1001genomes.org/accessions.html, accessed 10^th^ February 2021). For each signature and each population group, we used Kruskal-Wallis tests to assess the null hypothesis that the two sets originate from the same continuous distribution. The null hypothesis was not rejected in any case. Note that a comparison was not possible for the Tajima’s *D* and Fay and Wu’s *H* data from Italy/the Balkans/the Caucasus, as there was insufficient data to populate the two groups.


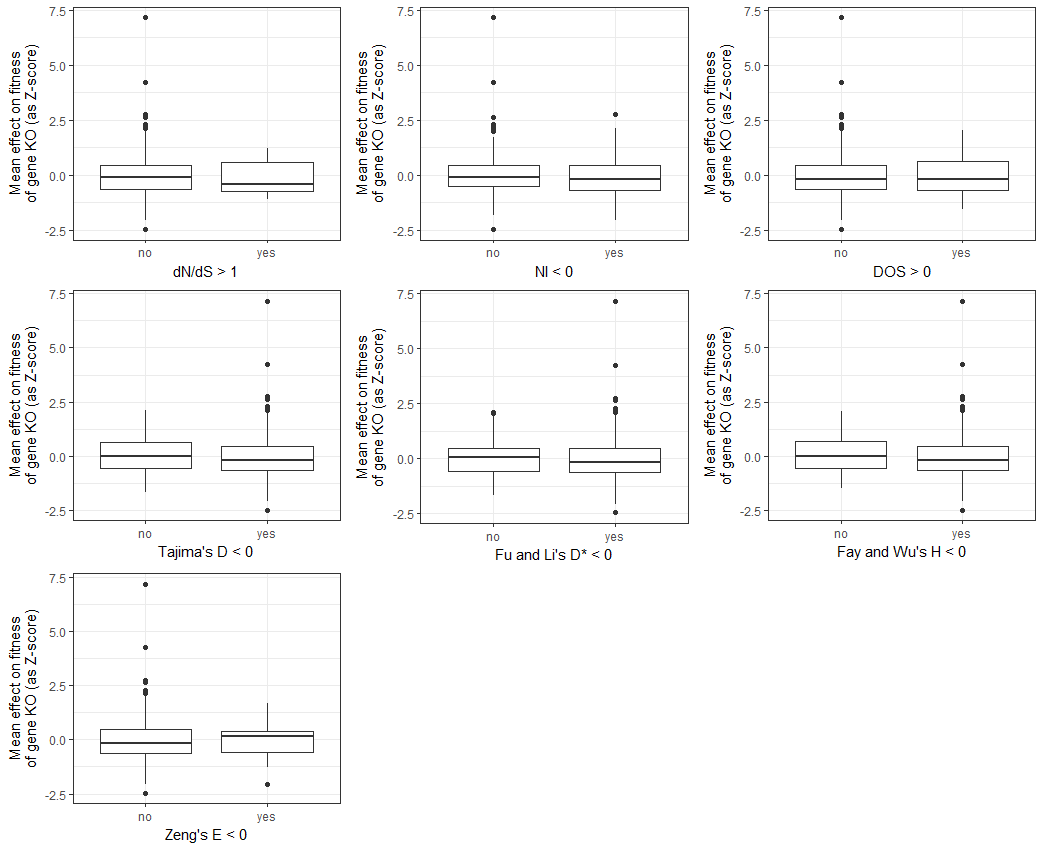


**Supplementary Figure 5.** The distribution of standardised fitness estimates does not significantly differ between genes with signatures of selection, and genes without the signature of selection.

Raw data for this figure is available in **Supplementary Tables 1** (selection) and **3** (fitness). The raw fitness estimates already follow a normal distribution and to that end further standardisation makes negligible difference; the boxplots in this figure are essentially identical to those shown in **Figure 3**. For seven different indices of sequence evolution, we used Kruskal-Wallis tests to assess the null hypothesis that the two sets originate from the same continuous distribution. The null hypothesis was not rejected for any measure: p = 0.453 (dN/dS), 0.409 (NI), 0.716 (DOS), 0.559 (Tajima’s *D*), 0.432 (Fu and Li’s *D**), 0.430 (Fay and Wu’s *H*), and 0.906 (Zeng’s *E*).


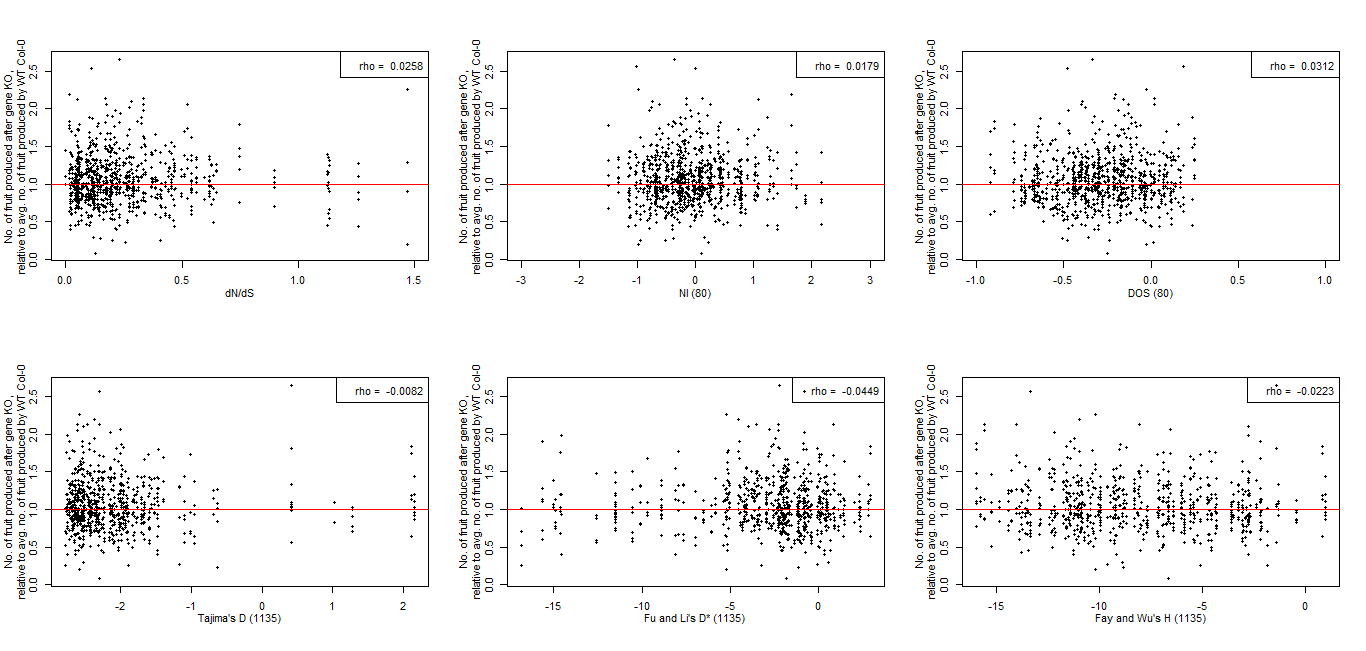


**Supplementary Figure 6.** Measures of sequence evolution poorly correlate with gene effect on fitness, after filtering to remove those with ‘area ratio’ > 1.5 (an indicator of the likelihood of multiple T-DNA insertions; see Materials and Methods).

This is contrary to the expectation that knocking out genes with strong evidence of positive or purifying selection will have a higher impact on fitness. This figure shows dN/dS, NI, DOS, *D*, *D** and *H* estimates for 861 datapoints, including all replicates, and representing 236 genes. Zeng’s *E*, which correlates strongly with *H* (see **Supplementary Text**), is not shown. The upper-right of each panel shows Spearman’s *rho* for the correlations of the each estimator with fitness, prior to correction for multiple testing. Although not plotted, a comparably insignificant correlation was found for Zeng’s *E* (*rho* = 0.02, p = 0.64).


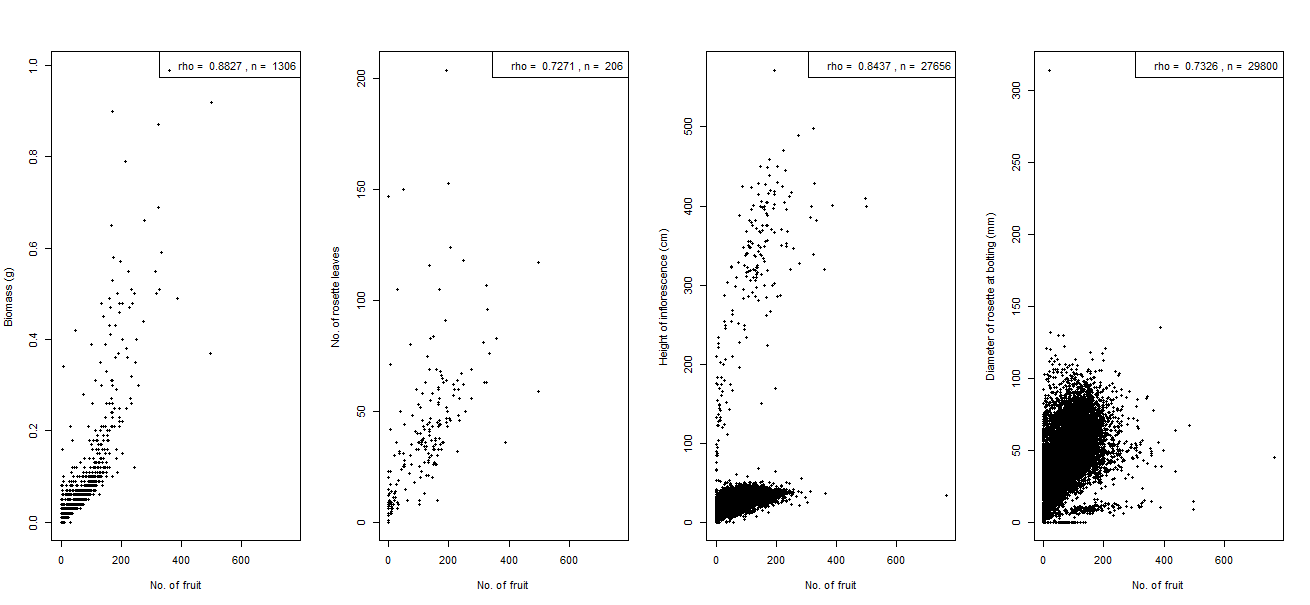


**Supplementary Figure 7.** Correlates of fruit count and four other phenotypes characterised for the unPAK knockout lines detailed in **Supplementary Table 3**: from left to right, biomass, height of the main stem inflorescence, number of rosette leaves, and the diameter of the rosette at bolting. The raw data for this figure is available in the ‘phenowide’ dataframe from the R package ‘unpakathon’.

**References**

Bush, S. J., Kover, P. X., & Urrutia, A. O. (2015). Lineage-specific sequence evolution and exon edge conservation partially explain the relationship between evolutionary rate and expression level in A. thaliana. *Mol Ecol, 24*(12), 3093-3106.

Gan, X., Stegle, O., Behr, J., Steffen, J. G., Drewe, P., Hildebrand, K. L., . . . Mott, R. (2011). Multiple reference genomes and transcriptomes for Arabidopsis thaliana. *Nature, 477*(7365), 419-423. doi:10.1038/nature10414
