# Supplementary material for: Contrasting gene‐level signatures of selection with reproductive fitness
Source: Mol Ecol. 2022 Jan 17;31(5):1515–26. doi: 10.1111/mec.16329 (PMC9304172; doi:10.1111/mec.16329)
Supplement: Supplementary file 3 — Text S1 [file MEC-31-1515-s002.docx]

**Contrasting gene-level signatures of selection with reproductive fitness**

Stephen J. Bush^1^, Courtney J. Murren^2^, Araxi O. Urrutia^3,4*^, Paula X. Kover^3*^

^1^ Weatherall Institute of Molecular Medicine, University of Oxford, Oxford, UK

^2^ Department of Biology, College of Charleston, Charleston, SC 29424

^3^ Milner Centre for Evolution, Department of Biology and Biochemistry, University of Bath, Bath, BA2 7AY, UK

^4^ Instituto de Ecologia, UNAM, Ciudad de Mexico 04510, Mexico

* Corresponding authors: p.x.kover@bath.ac.uk, a.urrutia@bath.ac.uk

**Supplementary Text**

This study estimates, using *Arabidopsis thaliana*, *Arabidopsis lyrata*, *Arabidopsis halleri* and *Thellungiella parvula*, seven gene-level substitution and polymorphism-based statistics commonly used to infer selection. These seven methods, collectively known as ‘neutrality tests’, include comparisons of nucleotide substitutions between species (dN/dS (1)), polymorphism within species (Tajima’s *D* (2), and Fu and Li’s *D** (3)), polymorphism both within and between species (Fay and Wu’s *H* (4) and Zeng’s *E* (5)), and those that combine substitution and polymorphism data, such as two derivatives of the McDonald-Kreitman test, the ‘neutrality index’ (NI) (6) and ‘direction of selection’ (DOS) (7), which are based on 2x2 contingency tables of fixed and polymorphic synonymous and non-synonymous sites (8). This supplementary text describes how the distribution of each test statistic can be interpreted to identify the signature of past selection, the discordance between test statistics, and the use of this dataset to identify candidate genes for positive selection. It also provides additional detail on the unPAK experimental methodologies used to generate the empirical estimates of gene effect on fitness, for contrast with these signatures of selection.

***Interpreting the results of neutrality tests***

The simplest and most commonly used method of identifying genes that have undergone past selection is **dN/dS**. This statistic calculates the ratio of non-synonymous (dN) to synonymous (dS) mutations at each site, from which positive (adaptive) selection is commonly inferred when dN/dS > 1 and negative (purifying) selection when dN/dS < 1 (9). Although commonly used, this threshold is particularly conservative as it averages over the whole coding sequence, thus averaging the effect of potentially opposing selective forces at different nucleotides. It is possible that positive selection is instead restricted only to specific regions of a gene, for example the antigen-recognition site of the human MHC locus, where specificity is maintained by pathogen-driven balancing selection (10). A criticism of dN/dS to infer adaptive evolution is that it assumes that repeated amino acid replacements at a limited set of codons are characteristic of positive selection in general, whereas adaptive phenotypes can result from many other (often untested) causes (11). Non-selective forces can also confound the interpretation of dN/dS by leaving genomic signatures consistent with positive selection, such as those arising from GC-biased gene conversion, a recombination-associated process which accelerates the fixation of guanine and cytosine alleles (12-14).

In contrast to dN/dS, the two McDonald-Kreitman-style tests, **NI** and **DOS**, both produce relatively symmetrical distributions that allow the inference of positive selection when NI < 0 or DOS > 0; and of purifying selection when NI > 0 or DOS < 0.

Other tests used in this study employ only polymorphism data, and distinguish between selective and non-selective interpretations. Among the methods based solely on within-species polymorphism one of the most common is **Tajima’s *D***. This statistic compares the number of segregating sites (those at which a polymorphism is found) with the nucleotide diversity at those sites, identifying either an excess (negative *D*) or dearth (positive *D*) of variants. Tajima’s *D* statistic can be interpreted as consistent with scenarios either of positive/purifying selection and/or population expansion (negative *D*), or balancing selection (when more than one allele is maintained in a population) and/or bottlenecking (positive *D*), respectively. However, as *D* was not intended to detect any particular deviation from a neutral model, it is particularly sensitive to both demographic and selective forces. Consequently, it has relatively low specificity for detecting positive selection alone.

To help distinguish between the selectionist and non-selectionist interpretations of a negative *D*, **Fay and Wu’s *H*** extends Tajima’s *D* test by incorporating data from the frequency spectrum of non-ancestral alleles, obtained from an outgroup. This allows the detection of any excesses of high-frequency SNPs compared to low- or intermediate-frequency variants. A negative *H* indicates an excess of high-frequency SNPs in a region of low overall nucleotide diversity (15). This is expected when a new, beneficial, allele is strongly selected as this causes a selective sweep. Sweeps reduce overall diversity in the region surrounding the selected allele by altering the frequency of nearby SNPs due to hitchhiking (16). Conversely, a positive *H* indicates a relative dearth of intermediate- and high-frequency SNPs (15). This is consistent with a scenario of purifying selection, whereby new (rare) mutations in a gene are deleterious and quickly purged, maintaining the ancestral state. Non-selective interpretations of *H* are also possible: population expansion following a recent bottleneck can also result in a relative excess of rare alleles (the number of nucleotide differences between two sequences being more strongly affected by a bottleneck than the number of segregating sites (17)), and hence a negative *H*.

In our dataset, the influence of non-selective forces on *A thaliana* was particularly apparent when estimating Fay and Wu’s *H*, which showed a whole genome distribution skewed towards negative values (see Figure 1 in the main text). While a selective interpretation of negative *H* is a recent selective sweep at a nearby locus, previous work has highlighted only one strong candidate for a species-wide sweep in *A. thaliana*: an approx. 300kb intrachromosomal transposition (18) for which the region of most extreme haplotype sharing, from 20.34 Mb to 20.49 Mb on chromosome 1, contains 50 genes (19) (of these 50 genes, three – AT1G54510, AT1G54520, and AT1G54920 – show evidence of positive selection on the basis of six of the seven tests, including dN/dS > 1; **Supplementary Table 1**). The whole genome skew to negative *H* can plausibly be attributed to the genomes of inbred species having, in general, longer haplotype lengths – in the case of *A. thaliana*, the haplotype affected by a selective sweep can be the entire chromosome. It is also important to note that when estimating *H*, the outgroup *A. lyrata*, is self-incompatible. Self-fertilising species such as *A. thaliana* have a lower effective recombination rate than out-crossing species (20), exacerbating the effect of background selection and so reducing levels of polymorphism (21) (although *A. thaliana* does appear to outcross occasionally (22)). As such, an excess of high-frequency SNPs in a region of low overall nucleotide diversity would be more apparent in *A. thaliana* not necessarily because these high-frequency SNPs were recently fixed but because nucleotide diversity is comparatively lower. This would also explain the strong agreement between Fay and Wu’s *H* and Zeng’s *E* (see Figure 1 in the main text), an additional test for selective sweeps discussed below.

**Zeng’s *E*** is based on the principle that should a large amount of variation be removed by a selective sweep, the power of the *D* and *H* tests can peak before fixation. This is because high-frequency variants only accumulate at the start of a sweep, concomitant with the loss of intermediate-frequency variants (5). Unlike *D*, which contrasts low- and intermediate-frequency variants, and *H*, which contrasts intermediate- and high-frequency variants, *E* contrasts low- and high-frequency variants. By doing so, *E* is particularly powerful at detecting the final, recovery, stage of a sweep – that is, after a beneficial allele has been fixed and neutral variants again re-accrue (5). *E* is calculated on the basis that the return to neutral expectations after a sweep is more rapid for low- compared to high-frequency variants, with negative values of *E* suggestive of a previous selective sweep (that is, where the selective event is relatively old, *E* not being especially sensitive to recent selection (23)). However, for similar reasons, *E* is also a sensitive test of population expansion: higher-frequency variants are the last to reach equilibrium after expansion (5).

Finally, **Fu and Li’s *D**** statistic uses intraspecific data to test for skew in the allele frequency spectrum. As with Tajima’s *D*, its expected value under a null model is 0, although *D** is more sensitive to the effects of selective sweeps. Fu and Li’s *D** statistic compares the number of segregating sites at which the variant is a singleton (that is, found only once in a sample) to the total number of variants between sequences (3). Although extremes of *D* and *D** are informative about both demographic and selective events, a relative excess of singletons would produce both a negative Fu and Li’s *D** and a negative Tajima’s *D*. This is particularly characteristic of a region immediately adjacent to a target of positive selection, one containing new mutations that have accumulated after a recent sweep (24), although can also arise from recent population expansion (which results in a relative excess of rare alleles) (23).

***Discordance between neutrality tests***

Although each of the above test statistics can be used to predict candidate genes for positive selection, we find little agreement between them (as did (25)). This might not be surprising since generally speaking, different methods are better at detecting selective events from different time periods (26), as discussed above. It is expected that sequence substitutions between species would reflect more ancient selective events, whereas methods examining variations in polymorphism rates would tend to detect the effects of recent and/or ongoing selection (27-29). A further source of discrepancies arises from the different sensitivity each method has to sources of bias, such as demographic parameters (30), although the extent of demographic sensitivity may only be known in a few systems. In addition, tests of deviation from neutrality typically only report outlier loci, which can suffer from high false positive rates (31).

Comparing the concordance among methods in the ranking of genes (see **Figure 1**, below) we found only marginal similarity in gene ranking among most estimators. For instance, the correlation between dN/dS and Tajima’s *D*, which are among the most widely used estimates, is a negligible Spearman’s *rho* = -0.02. Only two estimators, Fay and Wu’s *H* and Zeng’s *E*, were strongly concordant (*rho* = -0.98), although as discussed above, these essentially measure the same thing.

Finally, in addition to different methods identifying different genes, we also observe little overlap in enriched Gene Ontology (GO) biological process terms (**Supplementary Table 2**).

***Identifying genes under positive selection***

The conservative set of 29 candidate genes (**Supplementary Table 1**), considered positively selected according to all seven neutrality tests, span a broad range of biological roles including development (e.g. *MAF1* and *PPR287*), the response to biotic stress (e.g. *LUP1* and *PSS1*) and abiotic stress (e.g. *CSN1* and *OTS1*), and the regulation of gene expression (e.g. *HAC12* and *NRPA1*). Functional associations of genes with signatures of positive selection are detailed below, with additional detail given in the endnotes.

**Genes considered positively selected by 6 or more measures (dN/dS > 1, DOS > 0 and NI < 0, in both cases using polymorphism data from 80 *Arabidopsis* accessions (32), and Tajima’s *D*, Fu and Li’s *D**, Fay and Wu’s *H* and Zeng’s *E* < 0).**

The 29 genes highlighted in yellow are considered positively selected by all 7 measures.

| **Gene symbol, via Ensembl** | **Function** | **Reference** |
| --- | --- | --- |
| APRR8 | cytokinin involved in light-dependent regulatory processes | (33) |
| ATRPAC43 | encodes an enzyme associated with photoautotrophic growth, but is functionally redundant | (34) |
| AT1G48740 (CP4) | member of the CUPULIFORMIS (CP) family, which comprises paralogues of ICU11, a component of the epigenetic machinery | (35) |
| AT1G50140 | unknown | n/a |
| AT1G79950 (RTEL1) | has multiple roles in preserving genome stability, including telomere homeostasis. Mutations in the human orthologue result in a multi-system developmental disorder, Hoyeraal-Hreidarsson syndrome | (36) |
| AT3G49630 | unknown | n/a |
| AT3G50590 (TWD40-1) | member of the transducin/WD40 repeat-like superfamily; has a potential role in subcellular trafficking | (37) |
| AT3G57220 | unknown | n/a |
| AT3G59040 (PPR287) | essential role in chloroplast biogenesis and function | (38) |
| AT3G59640 (PSS1) | enhances disease resistance in transgenic soybean plants (to the fungal pathogen *Fusarium virguliforme*, the causative agent of sudden death syndrome) | (39) |
| AT3G62620 | unknown | n/a |
| AT5G65400 | predicted to be a peroxisome-targeting signal (peroxisomes having numerous essential developmental roles) | (40) |
| BOR5 | putative boron transporter (boron is an essential micronutrient but toxic in excess; numerous transporters are involved in homeostasis) | (41) |
| CDS5 | required for phospholipid homeostasis and is negatively involved in hyperosmotic stress tolerance | (42) |
| CSN1 | encodes a subunit of the COP9 signalosome, a multifunctional protein complex with essential developmental functions | (43) |
| CYP76C7 | encodes a member of a metabolically-associated cytochrome P450 family; the locus of this gene is within a segmental duplication | (44) |
| DAPF | key member of the lysine biosynthesis pathway | (45) |
| FBL17 | essential for progression through the second mitosis during pollen development | (46) |
| FTSH12 | function unclear, although homozygous knockout results in embryonic defects | (47) |
| GPX5 | member of the glutathione peroxidase family of enzymes which protect against oxidative damage | (48) |
| HAC12 | a p300/CBP-type HAT domain gene, mutations in which are known to affect flowering time | (49-51) |
| LUP1 | an enzyme that converts oxidosqualene to pentacyclic triterpenes (a class of compounds implicated in the pharmacological effects of many plants used in folk medicine) | (52, 53) |
| MAF1 (FLM) | homologue of FLC (‘flowering locus C’), a floral repressor critical for plant survival during environmental stresses | (54-56) |
| NRPA1 | subunit of RNA polymerase I | (57) |
| NUP93B | interacts with the nuclear pore complex, which facilitates nucleocytoplasmic transport – a critical process for numerous cellular activities | (58) |
| OTS1 (ULP1D) | acts redundantly (with ULP1C) as a positive regulator of growth, with a role in the osmotic stress response | (59, 60) |
| PERK15 | orthologous to a gene that in *Brassica rapa* (Chinese cabbage) confers resistance to downy mildew | (61) |
| PRN1 | regulates seed germination and early seedling development | (62) |
| PTEN2B | encodes a dual phosphatase, associated with modulating signalling pathways controlling growth, metabolism and apoptosis. Is differentially regulated in response to environmental constraints including salinity and osmotic stress | (63) |
| SAC3A | putative mRNA export factor highly co-expressed with the developmentally-associated PRP4KA (mutations in which result in atypical rosettes, reduced branching, and late flowering) | (64) |
| SRF5 | segmentally duplicated member (along with SRF4) of the STRUBBELIG-RECEPTOR family of receptor kinases; involved in regulating many aspects of the cell cycle | (65) |
| SRS1 | homologue of SHI, which represses gibberellin responses (a hormone that mediates the transition to new developmental phases) | (66) |
| SULTR1;1 and SULTR1;2 | primarily involved in importing sulphate from the environment into the root | (67, 68) |
| TSO1 | negative regulator of MYB3R1, which acts to coordinate cell proliferation with differentiation in the shoot and root | (69) |

**Other genes considered positively selected, having at minimum dN/dS > 1**

| **Gene symbol, via Ensembl** | **Function** | **Reference** |
| --- | --- | --- |
| CLE41 | encodes one of several signalling peptides known as TDIF (tracheary element differentiation inhibitory factor), part of a complex regulatory network governing cambium development^1^ | (70) |
| CYCT1;2 | a cyclin-T protein, critical to regulating eukaryotic cell cycle progression, although exclusively expressed in anthers^2^ | (71) |
| ESFL10 (which may be re-annotated^3^ as AtPCP-Bα) | key regulator of the ‘hydration checkpoint’ in the establishment of pollen-stigma compatibility | (72) |
| MYB1 | transcription factor that coordinates cell signalling during salt stress | (73) |
| PSBK | core component of photosystem II with expression strongly correlated with water deficiency | (74) |
| PSY2 | rate-limiting enzyme in the carotenoid biosynthetic pathway, which is regulated by light levels | (75) |
| RCI2A | induced in response to cold, dehydration, salt stress and abscisic acid (a key stress hormone in plants) | (76) |
| RTFL18 | involved in the development of leaf primordia | (77) |
| SCRL5  and  LCR60 | defensin-like, i.e. members of one of the largest and most diverse family of pathogenesis-related genes | (78) |
| WRKY68 | negative regulator of innate immunity in rice | (79) |

***unPAK database methodologies: empirical measurements of gene effect on fitness***

To conduct large scale phenotyping of plants at reproductive maturity in common conditions, unPAK (80) uses a distributed phenotyping approach. The distributed network approach allows for parallel assessment in a replicate screening design of the Salk T-DNA mutant lines (81) for quantitative traits. Prior to experimentation, individual lines are bulked in a common growth environment and maternal plants of Salk lines screened for insert number (for details, see (80, 82)). As block effects are routinely observed in plant ecology and agricultural studies, the robust design includes the use of natural accession phytometers to account for microenvironmental variation in addition to wildtype lines as controls. Replicate controls are planted in each tray within each randomized block design screen. Phytometers are then available for use as scalars (e.g. Z-score transformations) to account for intra and inter-chamber microenvironmental variation. For a robust distributed reproducible design, rigorous protocols for plant growing conditions and phenotyping were implemented. Training and re-training in protocols at each phenotyping institution was conducted by the project manager. For data to be included in the unPAK database, screens included standardized pot sizes, light regime, watering regime, potting mix, seed cold treating methods and phenotyping methods (for further details, see (80)). Protocols for growing conditions and phenotyping materials are available both in written format and as videos, and have been published as supplementary materials (e.g., Data S1 of (80)). After phenotyping, all plants are retained for future review or additional analyses. Replicate plants are grown for each line within replicate chambers – where intra-accession differences are observed for the same set of lines across screens at different institutions (example, in Figure 2 of (80)). Reproductive output is defined as total fruit (siliques) produced per plant which produce seeds. Re-counting of reproductive output by multiple research interns consistently demonstrates the reproducibility of quantitative measures and was supported by regular phenotyping spot-checks by the staff scientist project manager. Data QA/QC was initially conducted by faculty at each phenotyping location followed by the project manager prior to input in the publicly available database. The majority of data available in the publicly available database are from screens in the common growth chamber conditions as examined here. Manipulated environmental treatments (if used) are denoted in the database with associated metadata and such treatments are easily subset. Course-based data collection are uniquely flagged in the database and researchers have the option to include only data collected by research interns or the entire data set. In the present study, only research intern data has been included. As a confirmatory validation of the data, **Figure 2** (see below) illustrates that the different Salk lines used in this study have significantly different fitness.

**Notes**

^1^ We can speculate that CLE41, with its role in cambium development, could be central to a process under selection. The cambium is located between the xylem and phloem and produces precursor cells for both, with TDIF (of which CLE41 encodes a component) governing phylum-xylem cross-talk and procambium stem cell fate (83). As the relative growth of xylem and phloem determines the length and strength of the plant stem, it is reasonable to believe this is subject to selection. Competition for solar energy drives stem elongation, but longer stems are both vulnerable to wind shear (mediated by the mechanical reinforcement of xylem cells) and constrain the efficiency of photosynthesis, as the products of photosynthesis must be transported back to the roots (mediated by phloem) (70).

^2^ Compared to other members of the CYCT family and although a core cell cycle gene, there is comparatively little functional information for CYCT1;2. This could reflect its absence from the commonly used Affymetrix ATH1 microarray (84). By contrast, the related genes CYCT1;4 and CYCT1;5 are important host targets of Cauliflower mosaic virus for transcriptional activation, knockouts of which result in complete viral resistance (85).

^3^ The gene ESFL10 (‘embryo surrounding factor 1-like protein 10’; AT5G61605) is one of several *Arabidopsis* genes that in the TAIR10 annotation are named for similarity to ESF1 (an essential regulator of pro-embryo patterning (86)), although there is little functional information available via Ensembl. Ensembl Plants v51 reports only one associated GO term (‘pollen-stigma interaction’) and only one substantive orthologue, a one-to-one relationship to the *A. lyrata* gene “fgenesh2_kg.8__2144__AT5G61605.1”, at > 80% reciprocal identity. On this basis, it is unclear in what manner this gene resembles ESF1, and so functional inferences based on this (automatically assigned) gene symbol alone can be misleading. Consistent with the GO term, an experimental characterisation of AT5G61605 classified this gene as a member of the PCP-B family (pollen coat protein B-class) of signalling proteins (72). These regulate the pollen-stigma interaction and thereby self-compatibility.

**Figures**


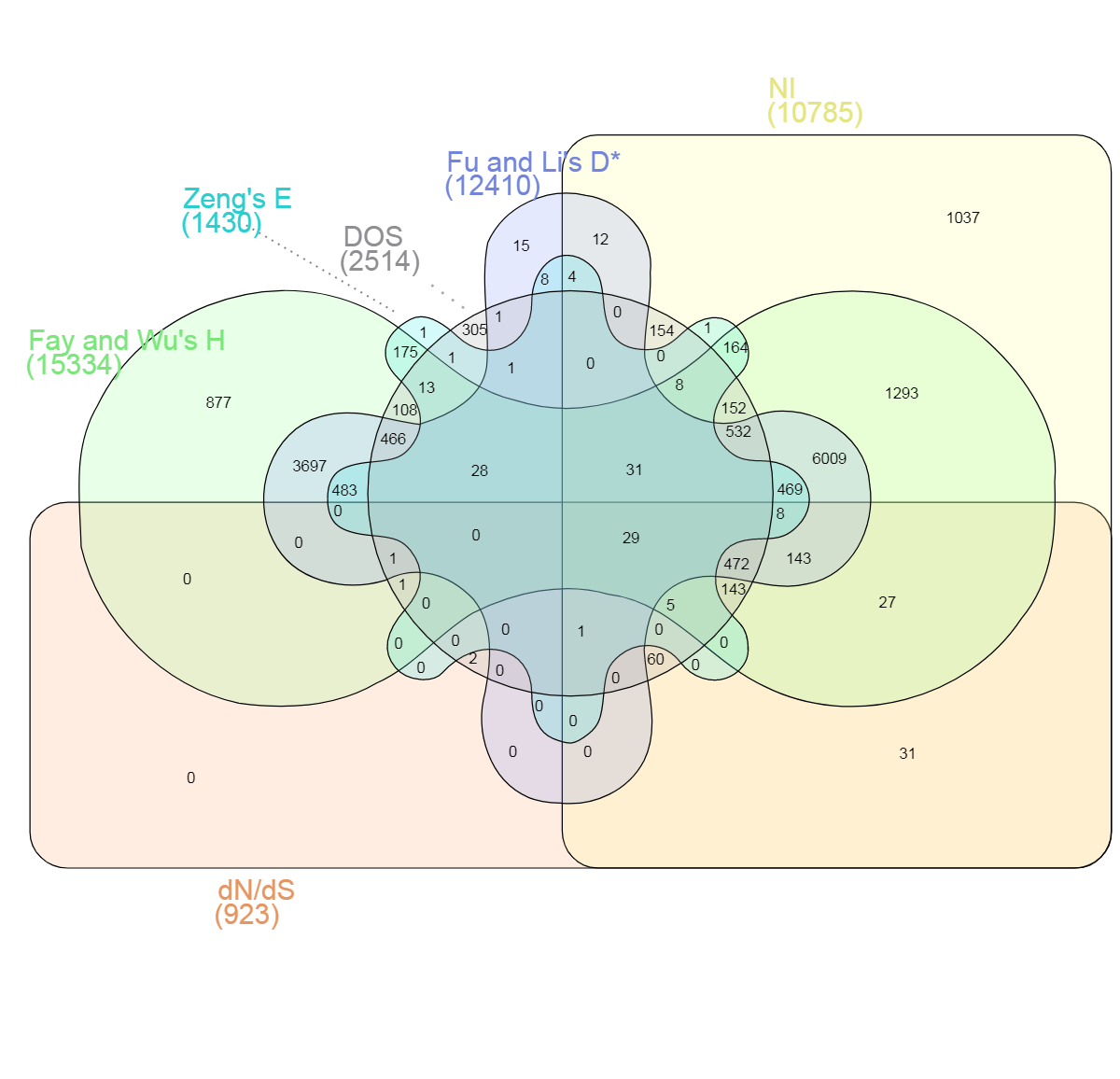


**Figure 1.** Minimal overlap between the number of genes considered positively selected using six different indices of sequence evolution calculated for up to 17,630 genes using the following common thresholds: dN/dS (> 1), NI (< 0), DOS (> 0), Tajima’s *D* (< 0), Fu and Li’s *D** (< 0), and Fay and Wu’s *H* (< 0). Raw data for this figure is available in **Supplementary Table 1**. The figure was created using InteractiVenn (87), which can plot a maximum of 6 sets. Note that those methods which draw on frequency spectrum data are particularly vulnerable to demographic effects, and that the ‘default’ threshold of 0 is not appropriate for predicting positively selected genes in *Arabidopsis*. Agnostic application of these thresholds has been used in this figure for illustrative purposes, to highlight the minimal overlap apparent between even large subsets.


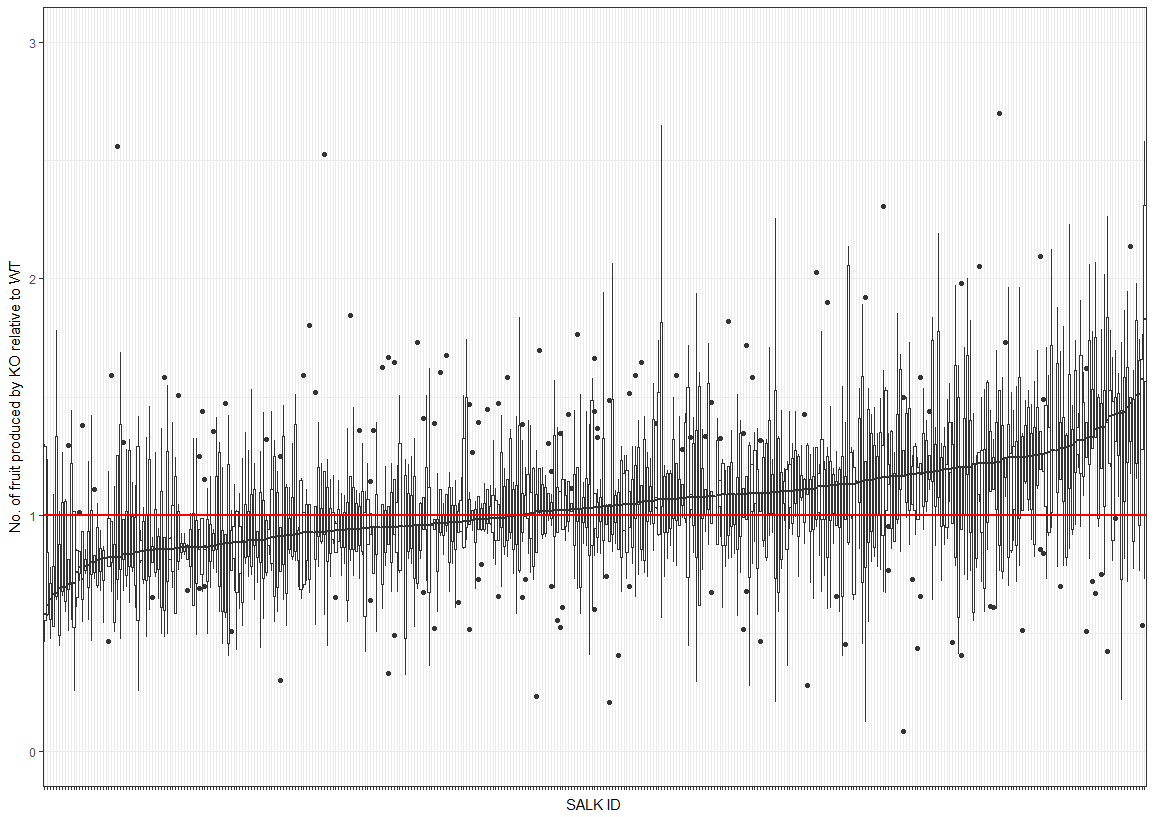


**Figure 2.** Differential effect of Salk line ID upon fitness.

The figure shows, for every replicate of each Salk line, the number of fruit produced by gene KO relative to the wildtype, Col-0 (i.e. gene effect on fitness). Boxes represent the interquartile range of these fitness estimates, with midlines representing the median. Upper and lower whiskers extend, respectively, to the largest and smallest values no further than 1.5x the interquartile range. Data beyond the ends of each whisker are outliers and plotted individually. Salk lines are ordered according to median fitness and are not otherwise labelled. The line y = 1 – indicative of no effect of KO upon fitness, relative to wildtype – is shown in red. While there is (as expected) a degree of noise in experimental observations, a clear signal is apparent. A Kruskal-Wallis test (non-parametric one-way ANOVA on ranks) assesses the null that the medians of all groups (i.e. SALK IDs) are equal. This null is rejected (p = 2.8 x 10^-6^) and so we can conclude that there is significant effect of Salk line ID upon fitness. The raw data illustrated in this figure is available in **Supplementary Table 3**.

**References**

1. Suzuki Y, Gojobori T. A method for detecting positive selection at single amino acid sites. Mol Biol Evol. 1999;16(10):1315-28.

2. Tajima F. Statistical method for testing the neutral mutation hypothesis by DNA polymorphism. Genetics. 1989;123(3):585-95.

3. Fu YX, Li WH. Statistical tests of neutrality of mutations. Genetics. 1993;133(3):693-709.

4. Fay JC, Wu CI. Hitchhiking under positive Darwinian selection. Genetics. 2000;155(3):1405-13.

5. Zeng K, Fu Y-X, Shi S, Wu C-I. Statistical tests for detecting positive selection by utilizing high-frequency variants. Genetics. 2006;174(3):1431-9.

6. Haldane JB. The estimation and significance of the logarithm of a ratio of frequencies. Annals of human genetics. 1956;20(4):309-11.

7. Stoletzki N, Eyre-Walker A. Estimation of the Neutrality Index. Molecular Biology and Evolution. 2011;28(1):63-70.

8. McDonald JH, Kreitman M. Adaptive protein evolution at the Adh locus in Drosophila. Nature. 1991;351(6328):652-4.

9. Hurst LD. The Ka/Ks ratio: diagnosing the form of sequence evolution. Trends in Genetics. 2002;18(9):486-7.

10. Ejsmond MJ, Radwan J. Red Queen Processes Drive Positive Selection on Major Histocompatibility Complex (MHC) Genes. PLoS Computational Biology. 2015;11(11):e1004627.

11. Hughes AL. Looking for Darwin in all the wrong places: the misguided quest for positive selection at the nucleotide sequence level. Heredity. 2007;99:364.

12. Ratnakumar A, Mousset S, Glemin S, Berglund J, Galtier N, Duret L, et al. Detecting positive selection within genomes: the problem of biased gene conversion. Philosophical transactions of the Royal Society of London Series B, Biological sciences. 2010;365(1552):2571-80.

13. Bolivar P, Mugal CF, Rossi M, Nater A, Wang M, Dutoit L, et al. Biased Inference of Selection Due to GC-Biased Gene Conversion and the Rate of Protein Evolution in Flycatchers When Accounting for It. Mol Biol Evol. 2018;35(10):2475-86.

14. Kostka D, Hubisz MJ, Siepel A, Pollard KS. The role of GC-biased gene conversion in shaping the fastest evolving regions of the human genome. Mol Biol Evol. 2012;29(3):1047-57.

15. Sterken R, Kiekens R, Coppens E, Vercauteren I, Zabeau M, Inze D, et al. A population genomics study of the Arabidopsis core cell cycle genes shows the signature of natural selection. The Plant cell. 2009;21(10):2987-98.

16. Smith JM, Haigh J. The hitch-hiking effect of a favourable gene. Genetical research. 1974;23(1):23-35.

17. Tajima F. The effect of change in population size on DNA polymorphism. Genetics. 1989;123(3):597-601.

18. Long Q, Rabanal FA, Meng D, Huber CD, Farlow A, Platzer A, et al. Massive genomic variation and strong selection in Arabidopsis thaliana lines from Sweden. Nature Genetics. 2013;45:884.

19. Clark RM, Schweikert G, Toomajian C, Ossowski S, Zeller G, Shinn P, et al. Common Sequence Polymorphisms Shaping Genetic Diversity in *Arabidopsis thaliana*. Science. 2007;317(5836):338.

20. Marais G, Charlesworth B, Wright SI. Recombination and base composition: the case of the highly self-fertilizing plant Arabidopsis thaliana. Genome Biology. 2004;5(7):R45.

21. Agrawal AF, Hartfield M. Coalescence with Background and Balancing Selection in Systems with Bi- and Uniparental Reproduction: Contrasting Partial Asexuality and Selfing. Genetics. 2016;202(1):313-26.

22. Weigel D, Nordborg M. Population Genomics for Understanding Adaptation in Wild Plant Species. Annual Review of Genetics. 2015;49(1):315-38.

23. Li H. A New Test for Detecting Recent Positive Selection that is Free from the Confounding Impacts of Demography. Molecular Biology and Evolution. 2010;28(1):365-75.

24. Jensen JD, Kim Y, DuMont VB, Aquadro CF, Bustamante CD. Distinguishing between selective sweeps and demography using DNA polymorphism data. Genetics. 2005;170(3):1401-10.

25. Paape T, Bataillon T, Zhou P, J. Y. Kono T, Briskine R, Young ND, et al. Selection, genome-wide fitness effects and evolutionary rates in the model legume Medicago truncatula. Molecular ecology. 2013;22(13):3525-38.

26. Akey JM. Constructing genomic maps of positive selection in humans: where do we go from here? Genome Research. 2009;19(5):711-22.

27. Oleksyk TK, Smith MW, Brien SJ. Genome-wide scans for footprints of natural selection. Philosophical Transactions of the Royal Society B: Biological Sciences. 2010;365(1537):185.

28. Sabeti PC, Schaffner SF, Fry B, Lohmueller J, Varilly P, Shamovsky O, et al. Positive natural selection in the human lineage. Science. 2006;312(5780):1614-20.

29. Kelley JL, Swanson WJ. Positive Selection in the Human Genome: From Genome Scans to Biological Significance. Annual review of genomics and human genetics. 2008;9(1):143-60.

30. Nielsen R. Statistical tests of selective neutrality in the age of genomics. Heredity. 2001;86(6):641-7.

31. Teshima KM, Coop G, Przeworski M. How reliable are empirical genomic scans for selective sweeps? Genome Res. 2006;16(6):702-12.

32. Cao J, Schneeberger K, Ossowski S, Gunther T, Bender S, Fitz J, et al. Whole-genome sequencing of multiple Arabidopsis thaliana populations. Nat Genet. 2011;43(10):956-63.

33. Brenner WG, Romanov GA, Kollmer I, Burkle L, Schmulling T. Immediate-early and delayed cytokinin response genes of Arabidopsis thaliana identified by genome-wide expression profiling reveal novel cytokinin-sensitive processes and suggest cytokinin action through transcriptional cascades. The Plant journal : for cell and molecular biology. 2005;44(2):314-33.

34. Haselier A, Akbari H, Weth A, Baumgartner W, Frentzen M. Two closely related genes of Arabidopsis encode plastidial cytidinediphosphate diacylglycerol synthases essential for photoautotrophic growth. Plant Physiology. 2010;153(3):1372-84.

35. Mateo-Bonmatí E, Esteve-Bruna D, Juan-Vicente L, Nadi R, Candela H, Lozano FM, et al. INCURVATA11 and CUPULIFORMIS2 Are Redundant Genes That Encode Epigenetic Machinery Components in Arabidopsis. The Plant Cell. 2018;30(7):1596-616.

36. Recker J, Knoll A, Puchta H. The *Arabidopsis thaliana* Homolog of the Helicase RTEL1 Plays Multiple Roles in Preserving Genome Stability. The Plant cell. 2014;26(12):4889.

37. Heard W, Sklenář J, Tomé DFA, Robatzek S, Jones AME. Identification of Regulatory and Cargo Proteins of Endosomal and Secretory Pathways in Arabidopsis thaliana by Proteomic Dissection. Mol Cell Proteomics. 2015;14(7):1796-813.

38. Lee K, Park SJ, Han JH, Jeon Y, Pai H-S, Kang H. A chloroplast-targeted pentatricopeptide repeat protein PPR287 is crucial for chloroplast function and Arabidopsis development. BMC plant biology. 2019;19(1):244.

39. Wang B, Sumit R, Sahu BB, Ngaki MN, Srivastava SK, Yang Y, et al. Arabidopsis Novel Glycine-Rich Plasma Membrane PSS1 Protein Enhances Disease Resistance in Transgenic Soybean Plants. Plant Physiology. 2018;176(1):865.

40. Reumann S, Quan S, Aung K, Yang P, Manandhar-Shrestha K, Holbrook D, et al. In-Depth Proteome Analysis of Arabidopsis Leaf Peroxisomes Combined with in Vivo Subcellular Targeting Verification Indicates Novel Metabolic and Regulatory Functions of Peroxisomes. Plant Physiology. 2009;150(1):125.

41. Yoshinari A, Takano J. Insights into the Mechanisms Underlying Boron Homeostasis in Plants. Frontiers in Plant Science. 2017;8(1951).

42. Hong Y, Yuan S, Sun L, Wang X, Hong Y. Cytidinediphosphate-diacylglycerol synthase 5 is required for phospholipid homeostasis and is negatively involved in hyperosmotic stress tolerance. The Plant Journal. 2018;94(6):1038-50.

43. Wang X, Kang D, Feng S, Serino G, Schwechheimer C, Wei N. CSN1 N-terminal-dependent activity is required for Arabidopsis development but not for Rub1/Nedd8 deconjugation of cullins: a structure-function study of CSN1 subunit of COP9 signalosome. Mol Biol Cell. 2002;13(2):646-55.

44. Höfer R, Boachon B, Renault H, Gavira C, Miesch L, Iglesias J, et al. Dual function of the cytochrome P450 CYP76 family from Arabidopsis thaliana in the metabolism of monoterpenols and phenylurea herbicides. Plant Physiology. 2014;166(3):1149-61.

45. Liu Y, Xie S, Yu J. Genome-Wide Analysis of the Lysine Biosynthesis Pathway Network during Maize Seed Development. PLoS ONE. 2016;11(2):e0148287-e.

46. Gusti A, Baumberger N, Nowack M, Pusch S, Eisler H, Potuschak T, et al. The Arabidopsis thaliana F-box protein FBL17 is essential for progression through the second mitosis during pollen development. PLoS ONE. 2009;4(3):e4780-e.

47. Wagner R, Aigner H, Pružinská A, Jänkänpää HJ, Jansson S, Funk C. Fitness analyses of Arabidopsis thaliana mutants depleted of FtsH metalloproteases and characterization of three FtsH6 deletion mutants exposed to high light stress, senescence and chilling. New Phytologist. 2011;191(2):449-58.

48. Milla MAR, Maurer A, Huete AR, Gustafson JP. Glutathione peroxidase genes in Arabidopsis are ubiquitous and regulated by abiotic stresses through diverse signaling pathways. The Plant Journal. 2003;36(5):602-15.

49. Deng W, Liu C, Pei Y, Deng X, Niu L, Cao X. Involvement of the Histone Acetyltransferase AtHAC1 in the Regulation of Flowering Time via Repression of FLOWERING LOCUS C in Arabidopsis. Plant Physiology. 2007;143(4):1660.

50. Han S-K, Song J-D, Noh Y-S, Noh B. Role of plant CBP/p300-like genes in the regulation of flowering time. The Plant Journal. 2007;49(1):103-14.

51. Servet C, Conde e Silva N, Zhou D-X. Histone Acetyltransferase AtGCN5/HAG1 Is a Versatile Regulator of Developmental and Inducible Gene Expression in Arabidopsis. Molecular Plant. 2010;3(4):670-7.

52. Segura MJ, Meyer MM, Matsuda SP. Arabidopsis thaliana LUP1 converts oxidosqualene to multiple triterpene alcohols and a triterpene diol. Organic letters. 2000;2(15):2257-9.

53. Ríos JL. Effects of triterpenes on the immune system. Journal of ethnopharmacology. 2010;128(1):1-14.

54. Ahn CS, Lee D-H, Pai H-S. Characterization of Maf1 in Arabidopsis: function under stress conditions and regulation by the TOR signaling pathway. Planta. 2019;249(2):527-42.

55. Ratcliffe OJ, Kumimoto RW, Wong BJ, Riechmann JL. Analysis of the Arabidopsis MADS AFFECTING FLOWERING gene family: MAF2 prevents vernalization by short periods of cold. The Plant cell. 2003;15(5):1159-69.

56. Ratcliffe OJ, Nadzan GC, Reuber TL, Riechmann JL. Regulation of flowering in Arabidopsis by an FLC homologue. Plant Physiology. 2001;126(1):122-32.

57. Ream TS, Haag JR, Pontvianne F, Nicora CD, Norbeck AD, Paša-Tolić L, et al. Subunit compositions of Arabidopsis RNA polymerases I and III reveal Pol I- and Pol III-specific forms of the AC40 subunit and alternative forms of the C53 subunit. Nucleic Acids Research. 2015;43(8):4163-78.

58. Tamura K, Fukao Y, Iwamoto M, Haraguchi T, Hara-Nishimura I. Identification and characterization of nuclear pore complex components in Arabidopsis thaliana. The Plant cell. 2010;22(12):4084-97.

59. Castro PH, Couto D, Freitas S, Verde N, Macho AP, Huguet S, et al. SUMO proteases ULP1c and ULP1d are required for development and osmotic stress responses in Arabidopsis thaliana. Plant molecular biology. 2016;92(1-2):143-59.

60. Conti L, Kioumourtzoglou D, O'Donnell E, Dominy P, Sadanandom A. OTS1 and OTS2 SUMO proteases link plant development and survival under salt stress. Plant Signal Behav. 2009;4(3):225-7.

61. Kim S, Song YH, Lee JY, Choi SR, Dhandapani V, Jang CS, et al. Identification of the BrRHP1 locus that confers resistance to downy mildew in Chinese cabbage (Brassica rapa ssp. pekinensis) and development of linked molecular markers. Theor Appl Genet. 2011;123(7):1183-92.

62. Lapik YR, Kaufman LS. The Arabidopsis cupin domain protein AtPirin1 interacts with the G protein alpha-subunit GPA1 and regulates seed germination and early seedling development. The Plant cell. 2003;15(7):1578-90.

63. Pribat A, Sormani R, Rousseau-Gueutin M, Julkowska MM, Testerink C, Joubès J, et al. A novel class of PTEN protein in Arabidopsis displays unusual phosphoinositide phosphatase activity and efficiently binds phosphatidic acid. The Biochemical journal. 2012;441(1):161-71.

64. Kanno T, Venhuizen P, Wen T-N, Lin W-D, Chiou P, Kalyna M, et al. PRP4KA, a Putative Spliceosomal Protein Kinase, Is Important for Alternative Splicing and Development in Arabidopsis thaliana. Genetics. 2018;210(4):1267-85.

65. Eyüboglu B, Pfister K, Haberer G, Chevalier D, Fuchs A, Mayer KFX, et al. Molecular characterisation of the STRUBBELIG-RECEPTOR FAMILY of genes encoding putative leucine-rich repeat receptor-like kinases in Arabidopsis thaliana. BMC plant biology. 2007;7(1):16.

66. Fridborg I, Kuusk S, Robertson M, Sundberg E. The Arabidopsis protein SHI represses gibberellin responses in Arabidopsis and barley. Plant Physiology. 2001;127(3):937-48.

67. Shibagaki N, Rose A, McDermott JP, Fujiwara T, Hayashi H, Yoneyama T, et al. Selenate-resistant mutants of Arabidopsis thaliana identify Sultr1;2, a sulfate transporter required for efficient transport of sulfate into roots. The Plant Journal. 2002;29(4):475-86.

68. Zhang B, Pasini R, Dan H, Joshi N, Zhao Y, Leustek T, et al. Aberrant gene expression in the Arabidopsis SULTR1;2 mutants suggests a possible regulatory role for this sulfate transporter in response to sulfur nutrient status. The Plant journal : for cell and molecular biology. 2014;77(2):185-97.

69. Wang W, Sijacic P, Xu P, Lian H, Liu Z. *Arabidopsis* TSO1 and MYB3R1 form a regulatory module to coordinate cell proliferation with differentiation in shoot and root. Proceedings of the National Academy of Sciences. 2018;115(13):E3045.

70. Oles V, Panchenko A, Smertenko A. Modeling hormonal control of cambium proliferation. PLOS ONE. 2017;12(2):e0171927.

71. Wang G, Kong H, Sun Y, Zhang X, Zhang W, Altman N, et al. Genome-Wide Analysis of the Cyclin Family in Arabidopsis and Comparative Phylogenetic Analysis of Plant Cyclin-Like Proteins. Plant physiology. 2004;135(2):1084.

72. Wang L, Clarke LA, Eason RJ, Parker CC, Qi B, Scott RJ, et al. PCP-B class pollen coat proteins are key regulators of the hydration checkpoint in Arabidopsis thaliana pollen-stigma interactions. New Phytol. 2017;213(2):764-77.

73. Wang T, Tohge T, Ivakov AA, Mueller-Roeber B, Fernie AR, Mutwil M, et al. Salt-Related MYB1 (SRM1) Coordinates Abscisic Acid Biosynthesis and Signaling During Salt Stress in Arabidopsis. Plant physiology. 2015.

74. Fan Y, Wang Q, Kang L, Liu W, Xu Q, Xing S, et al. Transcriptome-wide characterization of candidate genes for improving the water use efficiency of energy crops grown on semiarid land. Journal of Experimental Botany. 2015;66(20):6415-29.

75. Rodriguez-Villalon A, Gas E, Rodriguez-Concepcion M. Phytoene synthase activity controls the biosynthesis of carotenoids and the supply of their metabolic precursors in dark-grown Arabidopsis seedlings. The Plant journal : for cell and molecular biology. 2009;60(3):424-35.

76. Medina J, Catala R, Salinas J. Developmental and stress regulation of RCI2A and RCI2B, two cold-inducible genes of arabidopsis encoding highly conserved hydrophobic proteins. Plant physiology. 2001;125(4):1655-66.

77. Ikeuchi M, Yamaguchi T, Kazama T, Ito T, Horiguchi G, Tsukaya H. ROTUNDIFOLIA4 Regulates Cell Proliferation Along the Body Axis in Arabidopsis Shoot. Plant and Cell Physiology. 2011;52(1):59-69.

78. Mondragon-Palomino M, Stam R, John-Arputharaj A, Dresselhaus T. Diversification of defensins and NLRs in Arabidopsis species by different evolutionary mechanisms. BMC evolutionary biology. 2017;17(1):255.

79. Peng Y, Bartley LE, Chen X, Dardick C, Chern M, Ruan R, et al. OsWRKY62 is a negative regulator of basal and Xa21-mediated defense against Xanthomonas oryzae pv. oryzae in rice. Molecular plant. 2008;1(3):446-58.

80. Rutter MT, Murren CJ, Callahan HS, Bisner AM, Leebens-Mack J, Wolyniak MJ, et al. Distributed phenomics with the unPAK project reveals the effects of mutations. The Plant journal : for cell and molecular biology. 2019;100(1):199-211.

81. O’Malley RC, Ecker JR. Linking genotype to phenotype using the Arabidopsis unimutant collection. The Plant Journal. 2010;61(6):928-40.

82. Rutter MT, Wieckowski YM, Murren CJ, Strand AE. Fitness effects of mutation: testing genetic redundancy in Arabidopsis thaliana. Journal of evolutionary biology. 2017;30(6):1124-35.

83. Hirakawa Y, Shinohara H, Kondo Y, Inoue A, Nakanomyo I, Ogawa M, et al. Non-cell-autonomous control of vascular stem cell fate by a CLE peptide/receptor system. Proceedings of the National Academy of Sciences. 2008;105(39):15208.

84. Beemster GTS, De Veylder L, Vercruysse S, West G, Rombaut D, Van Hummelen P, et al. Genome-Wide Analysis of Gene Expression Profiles Associated with Cell Cycle Transitions in Growing Organs of Arabidopsis. Plant physiology. 2005;138(2):734.

85. Cui X, Fan B, Scholz J, Chen Z. Roles of *Arabidopsis* Cyclin-Dependent Kinase C Complexes in Cauliflower Mosaic Virus Infection, Plant Growth, and Development. The Plant Cell. 2007;19(4):1388.

86. Costa LM, Marshall E, Tesfaye M, Silverstein KA, Mori M, Umetsu Y, et al. Central cell-derived peptides regulate early embryo patterning in flowering plants. Science. 2014;344(6180):168-72.

87. Heberle H, Meirelles GV, da Silva FR, Telles GP, Minghim R. InteractiVenn: a web-based tool for the analysis of sets through Venn diagrams. BMC bioinformatics. 2015;16(1):169-.
